# ZTF-8 Interacts with the 9-1-1 Complex and Is Required for DNA Damage Response and Double-Strand Break Repair in the *C. elegans* Germline

**DOI:** 10.1371/journal.pgen.1004723

**Published:** 2014-10-16

**Authors:** Hyun-Min Kim, Monica P. Colaiácovo

**Affiliations:** Department of Genetics, Harvard Medical School, Boston, Massachusetts, United States of America; Stanford University Medical Center, United States of America

## Abstract

Germline mutations in DNA repair genes are linked to tumor progression. Furthermore, failure in either activating a DNA damage checkpoint or repairing programmed meiotic double-strand breaks (DSBs) can impair chromosome segregation. Therefore, understanding the molecular basis for DNA damage response (DDR) and DSB repair (DSBR) within the germline is highly important. Here we define ZTF-8, a previously uncharacterized protein conserved from worms to humans, as a novel factor involved in the repair of both mitotic and meiotic DSBs as well as in meiotic DNA damage checkpoint activation in the *C. elegans* germline. *ztf-8* mutants exhibit specific sensitivity to γ-irradiation and hydroxyurea, mitotic nuclear arrest at S-phase accompanied by activation of the ATL-1 and CHK-1 DNA damage checkpoint kinases, as well as accumulation of both mitotic and meiotic recombination intermediates, indicating that ZTF-8 functions in DSBR. However, impaired meiotic DSBR progression partially fails to trigger the CEP-1/p53-dependent DNA damage checkpoint in late pachytene, also supporting a role for ZTF-8 in meiotic DDR. ZTF-8 partially co-localizes with the 9-1-1 DDR complex and interacts with MRT-2/Rad1, a component of this complex. The human RHINO protein rescues the phenotypes observed in *ztf-8* mutants, suggesting functional conservation across species. We propose that ZTF-8 is involved in promoting repair at stalled replication forks and meiotic DSBs by transducing DNA damage checkpoint signaling via the 9-1-1 pathway. Our findings define a conserved function for ZTF-8/RHINO in promoting genomic stability in the germline.

## Author Summary

Proper response to DNA damage and repair of DNA double-strand breaks (DSBs) is important to maintain genomic integrity and promote both accurate chromosome segregation and tumor suppression. Here we define the roles of a previously uncharacterized and conserved protein, ZTF-8, which is required for proper DNA damage checkpoint activation as well as DSB repair. Specifically, we provide a direct demonstration that ZTF-8 participates in both mitotic and meiotic DSB repair and in the meiotic DNA damage checkpoint via interacting with the 9-1-1 complex in the *C. elegans* germline. We propose that ZTF-8 is involved in promoting repair at blocked replication fork sites and meiotic DSBs in part by transducing DNA damage checkpoint signaling via the 9-1-1 DNA damage response complex.

## Introduction

Genome instability is a hallmark of cancer cells and a critical feature that enables tumor progression. Instability allows cells to break and reform chromosomes, generate new oncogene fusions, inactivate tumor suppressor genes, amplify drug resistance genes, and therefore increase their malignancy. This whole progression often accompanies the disruption of DNA repair genes as the failure in DNA repair permits an increased rate of chromosome breakage and mutagenesis [1]. For example, many mutations involved in DNA repair genes have been linked to the progression of diverse cancers including breast, ovarian, and skin cancer, as well as leukemia and lymphomas. These include germline mutations in breast cancer susceptibility 1 (*BRCA1*), *BRCA2*, *BRIP1*, *RAD50*, the Nijmegen breakage syndrome *NBS1* gene and the Fanconi anemia *FA* genes [2]. Germline defects in three known RecQ helicases cause defined genetic disorders associated with cancer predisposition and/or premature aging. These include Bloom's, Werner's and Rothmund–Thomson syndromes, which are caused by defects in the *BLM*, *WRN* and *RECQ4* genes, respectively [3]–[5]. Considering that many germline mutations in DNA repair genes specifically involved in double-strand break repair (DSBR) are linked to tumor progression [2], and that failure to properly repair programmed meiotic DSBs can impair chromosome segregation, understanding DSBR at a molecular and cellular level, in a genetically tractable multicellular system, is of extreme importance.

Studies in the yeasts *S. cerevisiae* and *S. pombe* revealed various gene functions required for the DNA damage checkpoint pathway [6]. Most DNA damage response (DDR) genes were identified through the genetic analysis of mutants defective in either the transcriptional or cell cycle arrest responses to DNA damage. The DNA damage checkpoint proteins in *S. pombe* include those encoded by several radiation-repair (*rad*) genes. A phosphatidylinositol kinase family in *S. pombe* is both structurally and functionally related to human ATM and ATR [7]. However, the lack of an apoptosis pathway in yeast and the high degree of conservation for known components of the DDR pathway between worms and humans have positioned the nematode *C. elegans* as an excellent genetic system to study DNA damage induced cell cycle arrest and apoptosis [8]–[11].

Here we have identified a role for ZTF-8, a protein conserved from worms through humans, in the repair of both mitotic and meiotic DSBs and in the activation of the pachytene DNA damage checkpoint in the *C. elegans* germline. We show that ZTF-8 localizes to both chromatin and the nucleolus. Changes in its subcellular localization in response to DNA damage, as well as its ATL-1- and ATM-1-dependent chromatin localization, support a role for ZTF-8 in DDR and DNA repair. Moreover, *ztf-8* mutants exhibit specific DNA damage sensitivity to γ-irradiation (γ-IR) and hydroxyurea (HU), and not to UV, nitrogen mustard (HN2) or camptothecin (CPT) treatment, suggesting a role in DSBR. This is further supported by the activation of an S-phase checkpoint and the accumulation of recombination intermediates during both mitotic and meiotic progression in *ztf-8* mutant germlines. However, while the S-phase checkpoint is intact, impaired meiotic DSBR progression partially fails to trigger the CEP-1/p53-dependent DNA damage checkpoint in late pachytene, also suggesting a role for ZTF-8 in DDR. This is further supported by the interaction of ZTF-8 with MRT-2, the *C. elegans* homolog of the Rad1 protein found in *S. pombe*, Drosophila, and mammals, and a member of the 9-1-1 DDR complex, and the impaired localization of HUS-1 onto chromatin in response to exogenous DSB formation in *ztf-8* mutants. Loss of ZTF-8 function resulted in partially impaired activation of germ cell apoptosis, a reduced brood size and the accumulation of RAD-51 foci, all of which were rescued in transgenic worms expressing human RHINO, suggesting that its functions are conserved between species. Taken together, our analysis supports a model in which ZTF-8 plays a role in repair at stalled replication forks and meiotic DSBs as well as in meiotic DNA damage checkpoint response via the 9-1-1 pathway.

## Results

### ZTF-8 is a previously uncharacterized and conserved protein

**Figure 1:**
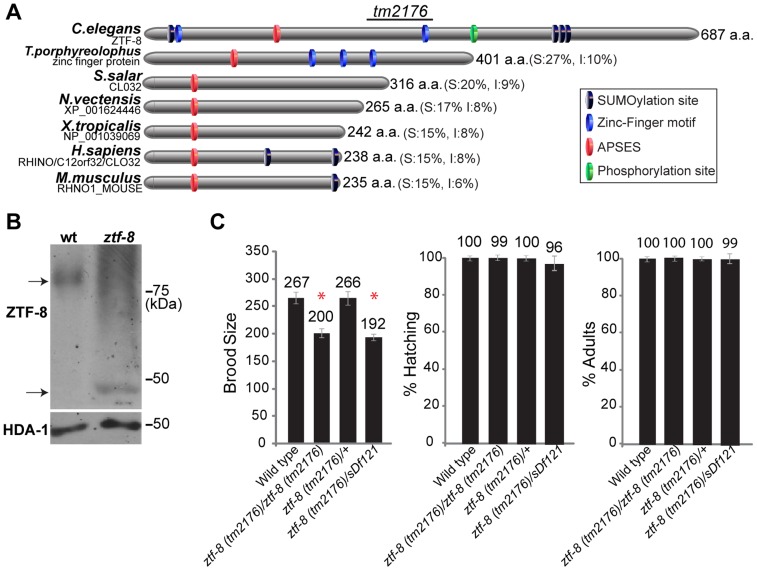
ZTF-8 is a conserved protein required for normal brood size. **A**. Schematic representation of the *C. elegans* ZTF-8 protein and predicted related proteins in *T.porphyreolophus*, *S.Salar, N.vectensis, X.tropicalis, H.sapiens and M.musculus*, indicating the region deleted in the *tm2176* mutant allele. Percent similarity and identity (S and I) compared to ZTF-8 are indicated in parentheses. Protein names and/or accession numbers are indicated. **B**. Western blot analysis comparing wild type and *ztf-8(tm2176)* mutant lysates probed with N-terminal anti-ZTF-8 and anti-histone deacetylase 1 (HDA-1; loading control) antibodies. **C**. Plate phenotypes of *ztf-8* mutants. Brood size, embryonic (shown as % hatching) or larval (% adults) survivals are scored among the progeny of worms of the indicated genotypes. Error bars represent standard error of the mean. n = 24 for each genotype. Asterisks indicate statistically significant reduction compared to wild type (P<0.0001 by the two-tailed Mann-Whitney test, 95% C.I.).

*ztf-8* (open reading frame ZC395.8) was identified in an RNAi-based screen performed as in [12] designed to find meiotic candidates among genes with germline-enriched expression in *C. elegans*. *ztf-8* encodes for a 687 amino acid protein that contains C2H2-type zinc-finger binding domains, and is highly conserved from worms to humans (Figure 1A; Figure S1). The ZTF-8 protein also carries a predicted DNA binding site (APSES) in its N-terminal region. The APSES motif has been reported to be required for the interaction between its putative mammalian homolog RHINO, a protein implicated in interacting with the Rad9-Rad1-Hus1 complex (9-1-1), and the ATR activator, TopBP1 [13], [14]. The high degree of conservation of the APSES motif throughout species supports a concurrent conservation of the DNA damage checkpoint machinery in different species.

### ZTF-8 is required for normal fertility and accurate meiotic chromosome segregation

The *ztf-8* deletion mutant (*tm2176*), obtained from the Japanese National Bioresource Project, carries a 524 base pair out-of-frame deletion encompassing most of exon 6 along with exons 7 through 11 (Figure 1A). This deletion results in a premature stop codon and the loss of a predicted zinc-finger motif, a predicted phosphorylation site, and three putative sumoylation sites. Analysis of wild type and *ztf-8* mutant lysates on Western blots, utilizing an affinity purified ZTF-8-specific N-terminal antibody, revealed that the protein migrates at a higher molecular weight than expected, and may therefore be undergoing some form of modification (77 kDa and 35 kDa were the expected protein sizes for wild type and the *ztf-8* mutant, respectively, Figure 1B). z*tf-8* mutants exhibit a 25% reduction in brood size compared to wild type, indicative of sterility (Figure 1C). Brood size was not significantly reduced in the heterozygotes, suggesting that *tm2176* is a recessive allele. *ztf-8(tm2176)* homozygous mutants do not exhibit any larval lethality suggesting that ZTF-8 does not have a role during larval development. However, homozygous mutants do exhibit weak, but significant, embryonic lethality (0.91% compared to 0.01% in wild type, P = 0.0078 by the two-tailed Mann-Whitney test, 95% C.I.) and high incidence of males (Him) phenotypes (0.26% compared to 0.03% in wild type, P = 0.0313), which taken together suggest a role in promoting accurate meiotic chromosome segregation. Finally, a *trans*-heterozygote for a deficiency encompassing the *ztf-8* locus (*ztf-8*(*tm2176*)/*sDf121*) did not exhibit a significant decrease in brood size (P = 0.6542) or an increase in either embryonic (P = 0.1823) or larval lethality (P = 0.0724) compared to *ztf-8(tm2176)* homozygous mutants, suggesting that *ztf-8(tm2176)* is likely a null (Figure 1C).

### ZTF-8 exhibits a dynamic localization throughout the germline

**Figure 2:**
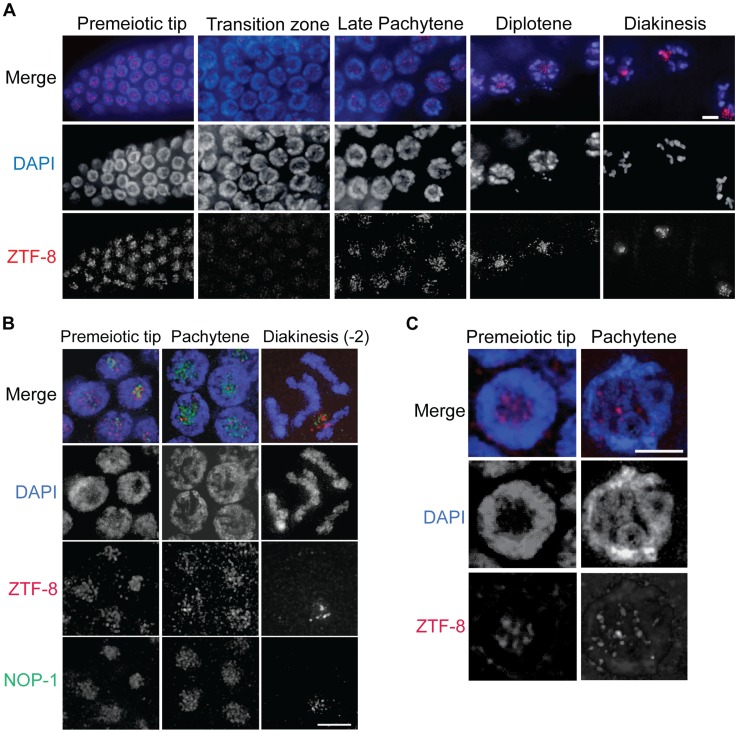
ZTF-8 is expressed in both mitotic and meiotic nuclei. **A**. Immunolocalization of ZTF-8 in whole mounted gonads utilizing a C-terminal peptide purified antibody against ZTF-8. Bar, 4 µm. **B**. Co-immunostaining with NOP-1, which encodes for a small nucleolar fibrillarin protein, reveals that ZTF-8 is enriched at the nucleolus. **C**. Localization of ZTF-8 at DAPI-stained chromosomes. At the premeiotic tip, 34% of ZTF-8 foci are localized to DAPI-stained chromosome. At late pachytene, 78% of ZTF-8 foci localize to DAPI-stained chromosomes (n = 102 nuclei at the premeiotic tip, 53 nuclei at pachytene, from 7–10 gonads). Bars, 2 µm.

To gain some insight into the function of ZTF-8 we examined its localization by immunostaining dissected wild type hermaphrodite gonads with an affinity purified ZTF-8 specific C-terminal antibody (Figure 2A). ZTF-8 signal is observed in mitotic nuclei at the distal tip (premeiotic tip). This signal is then reduced upon entrance into meiosis (leptotene/zygotene stages = transition zone) and remains weak through the mid-pachytene stage. However, the ZTF-8 signal increases once again in late pachytene nuclei and persists through late diakinesis oocytes. This dynamic pattern of expression suggests regulation of ZTF-8 during meiotic prophase.

At a higher resolution, ZTF-8 signal is observed as foci both on chromosomes as well as in the nucleolus (Figure 2B). Specifically, 34% of ZTF-8 foci at the premeiotic tip, and 78% at late pachytene, localize to DAPI-stained chromosomes, with the remaining foci being localized to the nucleolus (Figure 2C, n = 102 nuclei at the premeiotic tip, and 53 nuclei at pachytene, from 7–10 gonads). ZTF-8 localization is also observed in gut and embryonic nuclei and this signal is specific since it is absent in *ztf-8* homozygous mutants (Figure S2). Taken together, these localization studies suggest both mitotic and meiotic roles for ZTF-8.

### The mitotic DNA damage checkpoint is activated in *ztf-8* germline nuclei

**Figure 3:**
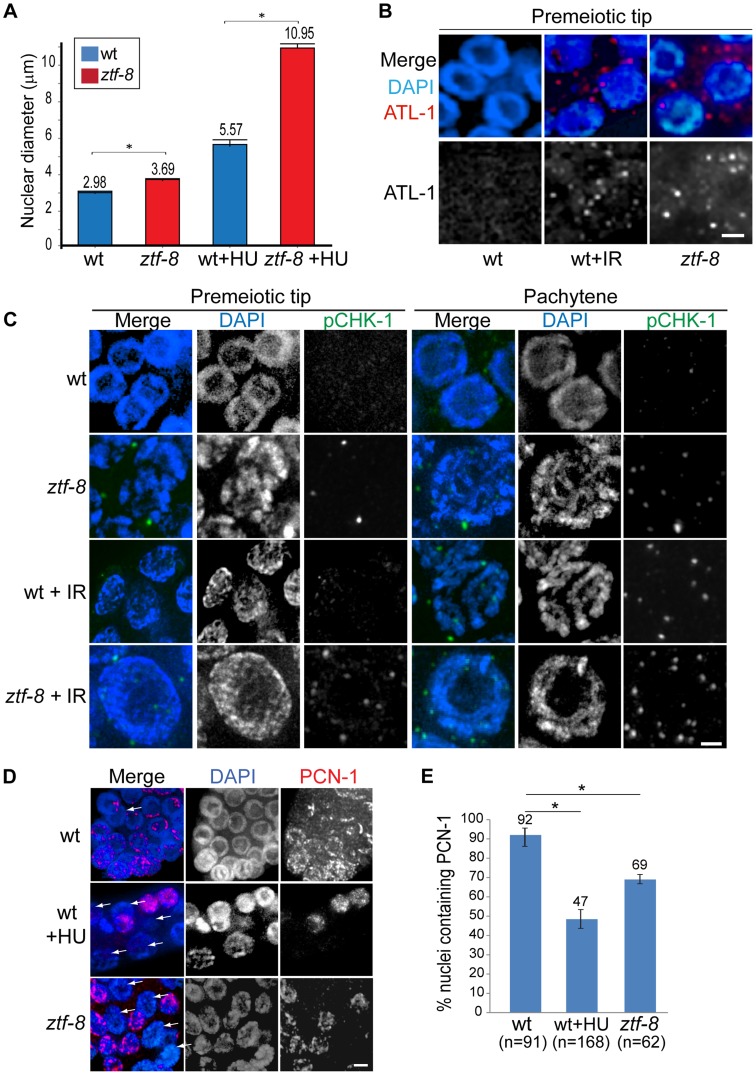
S-phase DNA damage checkpoint activation is intact in *ztf-8* mutants. **A**. *ztf-8* mutants exhibit enlarged nuclear diameters at the premeiotic tip. Asterisks indicate statistical significance by the two-tailed Mann-Whitney test, 95% C.I., P<0.0001 without HU treatment and P<0.0001 in 20 mM HU containing NGM media. n = 178, 170, 80, 81 for wt, *ztf-8*, wt+HU, and *ztf-8*+HU, respectively. **B**. Immunolocalization of ATL-1 in premeiotic tip nuclei of germlines from IR or non-IR treated wild type worms and *ztf-8* mutants. **C**. Immunostaining for phospho CHK-1 (pCHK-1) of germline nuclei at the indicated stages. Bar, 2 µm. **D**. 69% of mitotic germline nuclei in *ztf-8* mutants exhibit PCN-1 signal, which marks nuclei in S-phase, compared to 93% of nuclei in wild type. Arrows indicate nuclei lacking PCN-1 signal. Wild type worms exposed to 5 mM HU were used as a control for S-phase arrest. Bar, 2 µm. **E**. Quantitation of the percentage of nuclei containing PCN-1 signal. Asterisks indicate statistical significance. P = 0.0002 for wild type and wild type+HU and P = 0.0088 for wild type and *ztf-8* mutants. Statistical tests by the two-tailed Mann-Whitney test, 95% C.I.

Either exposure to genotoxic agents or DNA replication stress can lead to checkpoint responses in the *C. elegans* germ line. Specifically, the replication-dependent S-phase checkpoint is activated in response to stress, such as that stemming from HU treatment, DNA damage and abnormal DNA structures [15], and results in transient S-phase arrest, which is characterized by a premeiotic tip exhibiting enlarged nuclear diameters in the *C. elegans* germline [16]. In *ztf-8* mutants, enlarged mitotic nuclei were observed at the premeiotic tip compared to wild type (Figure 3A). Activation of the DNA damage checkpoint in *ztf-8* mutants is further supported by the elevated levels of ATL-1 (ATR homolog) and phosphorylated CHK-1 (pCHK-1) observed in these nuclei even without γ-IR exposure (Figure 3B and 3C). Given that ATL-1 is recruited to stalled replication fork sites [16], ZTF-8 is likely required for repair at stalled replication forks. This is supported by the further increase in nuclear diameter observed among mitotic nuclei at the premeiotic tip in the mutants following treatment with HU (3 fold induction in *ztf-8* mutants compared to 1.9 fold in wild type), a ribonucleotide reductase inhibitor which blocks DNA synthesis by preventing expansion of the dNTP pool and results in replication fork stalling (Figure 3A). Lower levels of PCN-1, the *C. elegans* ortholog of mammalian PCNA, in HU treated worms (Figure 3D and 3E) is further evidence of an S-phase arrest, consistent with studies in human cells where PCNA is absent from S-phase nuclei following HU treatment [17]. PCN-1 signal was observed only in 69% of mitotically dividing nuclei in *ztf-8* mutants compared to 92% in wild type, suggesting that slowing down S-phase in response to nucleotide depletion prevents association of PCN-1 onto replication sites. No significant difference is observed between *ztf-8* mutants and wild type with markers for G2/M and mitosis such as CDK-1 phospho-TYR15 and phospho-histone H3 (pSer10), respectively (Figure S3). Altogether, these observations indicate that there is activation of the S-phase checkpoint resulting in cell cycle arrest in the *ztf-8* mutants and that ZTF-8 function may be required for repair at stalled replication forks.

### *ztf-8* mutants are hypersensitive to DSBs induced by γ-IR and HU treatment

**Figure 4:**
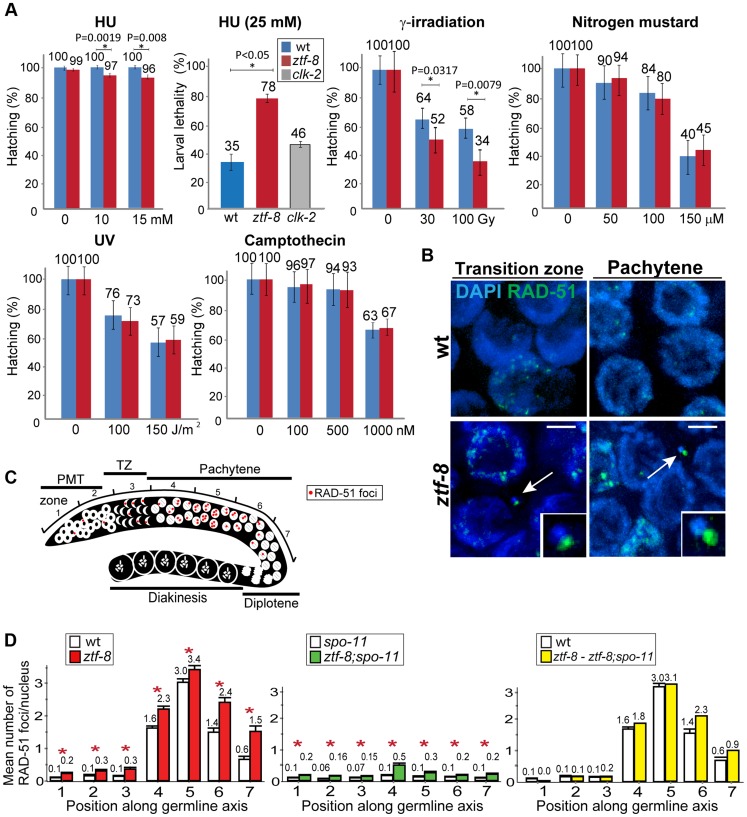
ZTF-8 is required for DNA repair in both mitotic and meiotic germline nuclei. **A**. Relative hatching or larval lethality of wild type, *ztf-8* and *clk-2* mutants after treatment with the indicated doses of hydroxyurea (HU), γ-irradiation, nitrogen mustard, UV and camptothecin. Asterisks indicate statistical significance; P values calculated by the two-tailed Mann-Whitney test, 95% C.I. **B**. IR-induced chromosome defects in the absence of *ztf-8*. Shown are representative images of nuclei at leptotene/zygotene (transition zone) and pachytene stages from wild type and *ztf-8* worms 18 hrs after γ-IR exposure (40 Gy). Chromosome fragments are indicated by arrows and depicted at higher magnification in the insets. Chromosome fragments were observed at the following frequencies: wt: 0/20 and *ztf-8* mutants: 6/20 gonads. Bars, 2 µm. **C**. Diagram of a *C. elegans* germline indicating the position of the zones scored for RAD-51 foci. **D**. Mean number of RAD-51 foci per nucleus. Histograms represent the quantitation of RAD-51 foci in germlines of the indicated genotypes. Quantitative analysis of RAD-51 foci depicted in Figure S4 is represented here as the mean number of RAD-51 foci observed per nucleus (y-axis) on each zone along the germline axis (x-axis) for indicated genotypes. To identify the levels of meiotic RAD-51 foci, mean number of RAD-51 foci at each zone in *ztf-8;spo-11* mutants was subtracted from *ztf-8* mutants. Error bars represent standard error of the mean. Asterisks indicate statistical significance.

To further examine the role of ZTF-8 in DNA damage repair, adult hermaphrodites were exposed to different types of DNA damage and embryonic lethality was monitored as in [18], [19] (Figure 4A). Exposure to HU, which results in a checkpoint-dependent cell cycle arrest, led to significant changes in hatching in *ztf-8* mutants compared to wild type (100% and 96%, respectively, at 15 mM). Also, *ztf-8* mutants showed increased larval lethality following HU exposure, further suggesting that *ztf-8* may be required for repair following collapse of stalled replication forks. *ztf-8* mutants exhibited reduced hatching frequencies compared to wild type following the induction of DSBs by γ-IR exposure. Specifically, only 52% and 34% hatching was observed among the progeny of *ztf-8* mutants exposed to either 30 or 100 Gy, respectively, compared to 64% and 58%, respectively, in wild type. Interestingly, in γ-IR exposed mutants we observed chromatin fragments with RAD-51 foci, which mark sites undergoing DSBR [20], present in nuclei from leptotene/zygotene to pachytene (Figure 4B). These observations strongly suggest that ZTF-8 is required for DSBR following γ-IR exposure. Exposure to HN2, which produces DNA interstrand crosslinks (ICLs), UV, which induces cyclobutane pyrimidine dimers, and CPT, which results in a single ended DNA double-strand break when collision of a replication fork occurs at the lesion, did not significantly reduce hatching levels in *ztf-8* mutants compared to wild type (Figure 4A). Taken together, these results indicate that the function of ZTF-8 in DNA repair exhibits a high degree of DNA damage specificity, being required for recovery from replication fork collapse and DSBR.

### ZTF-8 is required for normal DSBR progression in both mitotic and meiotic germline nuclei

**Figure 5:**
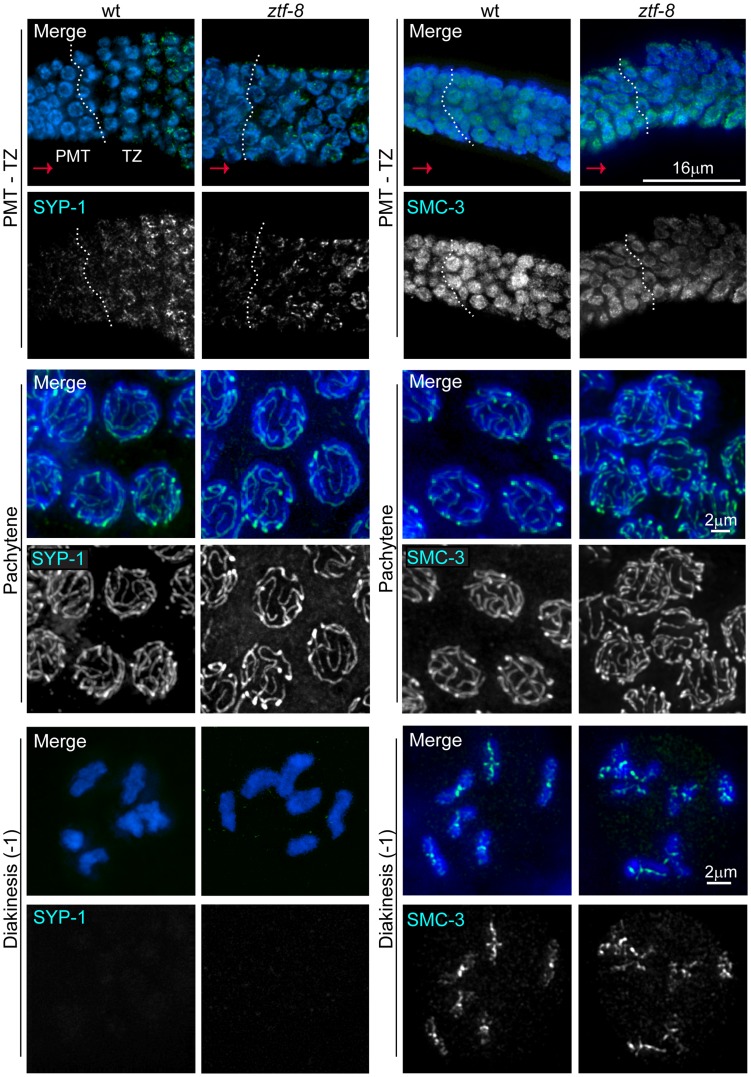
ZTF-8 is dispensable for axis morphogenesis and chromosome synapsis. Analysis of whole-mounted germlines from wild type and *ztf-8* mutants co-stained with DAPI (blue) and either SYP-1 (green) or SMC-3 (green), reveals that axis morphogenesis, and chromosome synapsis, are indistinguishable from wild type. Images show premeiotic tip (PMT)/transition zone (TZ), pachytene, and diakinesis nuclei where chromosomes initiate synapsis, are fully synapsed, and undergo SC disassembly, respectively. Progression from PMT to TZ is observed from left to right, as indicated by the red arrows; dotted white vertical lines indicate the boundary between the PMT and TZ. As in wild type, SYP-1 signal is observed associated with chromosomes from transition zone through most of diakinesis and is no longer present in the last oocyte prior to the spermatheca (−1 oocyte) in the mutants (20/20 gonads). Immunolocalization of SMC-3 reveals normal axis morphogenesis throughout meiotic progression in *ztf-8* mutants compared to wild type, with signal observed associated with chromosomes until the end of diakinesis (20/20 gonads).

To determine whether ZTF-8 is required for DSBR in both mitotic and meiotic nuclei, levels of RAD-51 foci were quantitated and compared between wild type and *ztf-8* germline nuclei (Figure 4C, 4D and Figure S4). Since nuclei are positioned in a temporal-spatial manner along the germline in *C. elegans*, proceeding in a distal to proximal orientation from mitosis into the various stages of meiotic prophase I, levels of RAD-51 foci were assessed both in mitotic (zones 1 and 2) and meiotic nuclei (zones 3–7). In wild type, a few mitotic RAD-51 foci were observed at zones 1 and 2, and they are mainly derived from single stranded DNA gaps formed at stalled replication forks or resected DSBs resulting from collapsed replication forks [21]. During meiotic prophase, SPO-11-dependent programmed meiotic DSBs are induced. Levels of RAD-51 foci start to rise at the transition zone (zone 3) and reach their highest levels at early to mid-pachytene (zones 4 and 5). As repair is completed, levels of RAD-51 foci are progressively reduced in late pachytene (zones 6 and 7). In *ztf-8* mutants, levels of RAD-51 foci were higher than those observed in wild type mitotic (20.7% of nuclei contained 1–3 RAD-51 foci compared to 7.8% for wild type in zones 1 and 2 combined, P<0.0001 by the two-tailed Mann-Whitney test, 95% C.I.) and meiotic germline nuclei (an average of 3.4 RAD-51 foci/nucleus were observed in *ztf-8* germlines at zone 5 compared to 3.0 for wild type; P = 0.0045). Higher levels of RAD-51 foci persisted through late pachytene in *ztf-8* mutants compared to wild type (2.4 RAD-51 foci/nucleus compared to 1.4, P = 0.0025, and 1.5 foci/nucleus compared to 0.6, P = 0.0081, in zones 6 and 7, respectively) suggesting either a delay in meiotic DSBR or an increase in the levels of DSBs formed during meiosis. This defect in DSBR does not stem from either impaired axis morphogenesis or chromosome synapsis since immunolocalization of either SMC-3, required for sister chromatid cohesion, or SYP-1, a central region component of the synaptonemal complex, was indistinguishable from wild type (Figure 5). To better distinguish the mitotic from the meiotic effects seen in DSBR we quantified the levels of RAD-51 foci in the germlines of *ztf-8;spo-11* double mutants, which lack the formation of meiotic programmed DSBs (Figure 4D). Elevated levels of RAD-51 foci were still present throughout the germline compared to *spo-11* single mutants, suggesting that DSBs of mitotic origin persist into the meiotic region in *ztf-8* mutants. To test if repair of programmed meiotic DSBs is also impaired in *ztf-8* mutants, we subtracted the number of foci of mitotic origin found in *ztf-8*; *spo-11* double mutants from the total number of RAD-51 foci observed in *ztf-8* single mutants (Figure 4D). Elevated levels of RAD-51 foci were still observed in the meiotic zones of *ztf-8* mutants compared to wild type (e.g. zones 6 and 7) indicating that meiotic DSBR is also impaired in *ztf-8* mutants contributing to the elevated levels of recombination intermediates detected in the germline. Taken together, these data support a role for ZTF-8 in promoting the normal progression of DSB repair in both mitotic and meiotic germline nuclei.

### The 9-1-1 mediated meiotic DNA damage response is impaired in *ztf-8* mutants

**Figure 6:**
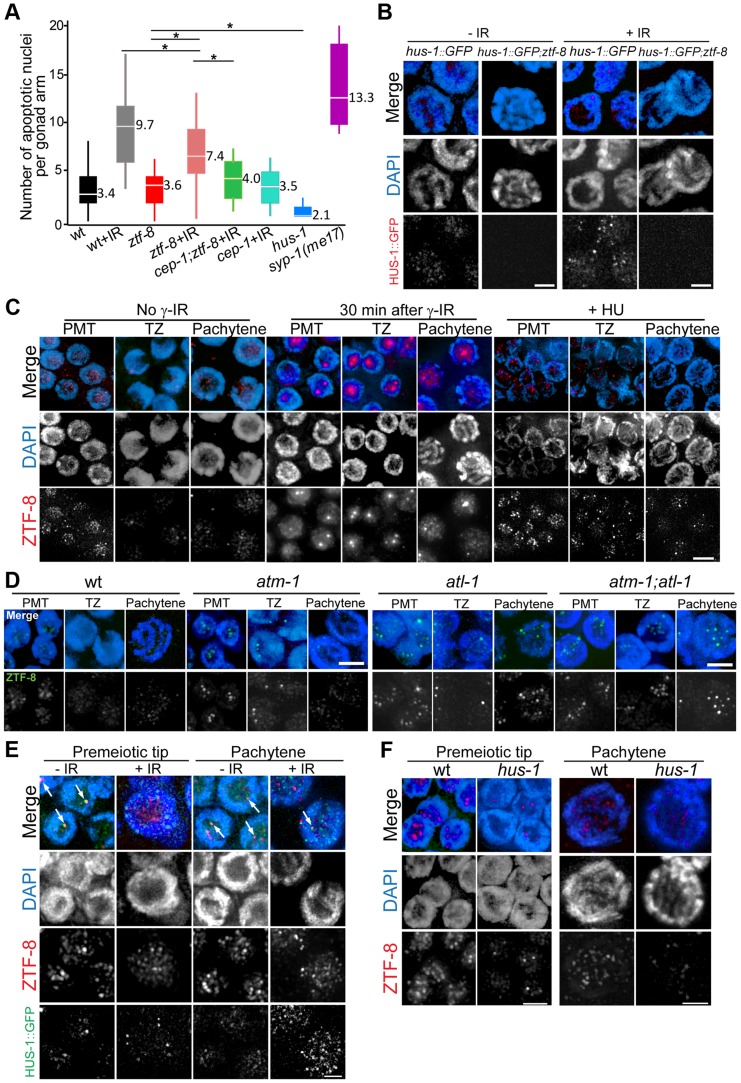
ZTF-8 is required for the 9-1-1 mediated meiotic DNA damage response and ZTF-8 localization requires both ATL-1 and ATM-1. **A**. Quantification of germline apoptosis in the indicated genotypes. +IR indicates treatment with γ-irradiation (80 Gy). *syp-1(me17)* is a synapsis-defective mutant with elevated germ cell apoptosis levels (MacQueen et al., 2002) utilized as a positive control. Asterisks indicate statistical significance. P = 0.004 for wt+IR and *ztf-8*+IR, P<0.0001 for all others, by the two-tailed Mann–Whitney test, 95% C.I. **B**. Expression of a HUS-1::GFP transgene in pachytene nuclei of either wild type or *ztf-8* mutants. **C**. Immunofluorescence images of nuclei stained with DAPI and anti-ZTF-8 prior to exposure to γ-IR, 30 minutes after γ-IR exposure (50 Gy), or exposed to 10 mM HU. Pre-meiotic tip (PMT), transition zone (TZ) and pachytene nuclei are shown. **D**. Immunofluorescence images of nuclei stained with DAPI and anti-ZTF-8 in wild-type, *atm-1*, *atl-1*, and *atm-1;atl-1* mutant backgrounds. **E**. Co-immunostaining with anti-ZTF-8 and HUS-1::GFP signal expressing worms either unexposed (-IR) or exposed (+IR) to 100 Gy of γ-irradiation. Germline nuclei being depicted are from worms 8 hours after γ-IR exposure to capture bright HUS-1::GFP foci as described in [8]. Arrows indicate overlapping ZTF-8 and HUS-1::GFP signals. **F**. Immunofluorescence images of nuclei stained with DAPI and anti-ZTF-8 in wild type and *hus-1* mutants. Bars, 2 µm.

Persistence of unrepaired DSBs can activate a DNA damage checkpoint resulting in increased apoptosis during late pachytene in the *C. elegans* germline [22]. Interestingly, the elevated levels of RAD-51 foci observed in *ztf-8* mutant germlines were not accompanied by increased levels of germ cell apoptosis in this mutant compared to wild type (P = 0.399, by the two-tailed Mann–Whitney test, 95% C.I., Figure 6A). Moreover, the levels of germ cell apoptosis were lower in *ztf-8* mutants compared to wild type following induction of exogenous DSBs by exposure to γ-irradiation (P = 0.004). These data suggest that either the DSBs marked by RAD-51 foci are repaired before nuclei are directed into an apoptotic fate or the DNA damage checkpoint machinery is impaired in *ztf-8* mutants. To examine this further, we used a HUS-1::GFP transgenic line and monitored the localization of this 9-1-1 DDR component in *ztf-8* mutants [8]. The weak HUS-1::GFP signal detected in *ztf-8* mutants compared to wild type, even after the induction of exogenous DSBs by γ-IR, suggests that the DNA damage checkpoint operating in late pachytene is impaired in *ztf-8* mutants (Figure 6B). However, the observation of higher levels of apoptosis in IR compared to non-IR treated *ztf-8* mutants suggests that activation of the late pachytene DNA damage checkpoint, while impaired, is still not fully abrogated in *ztf-8* mutants (Figure 6A, P<0.0001). In fact, the level of apoptosis observed in *ztf-8* mutants is higher than that in a *hus-1(op241)* mutant, which is required for CEP-1/p53-dependent DNA damage-induced apoptosis (Figure 6A, P<0.0001, [8]) suggesting only a partial reduction in the activation of germ cell apoptosis. Consistent with previous observations, the level of apoptosis observed in irradiated *ztf-8* mutants is significantly reduced in *cep-1*;*ztf-8* mutants (Figure 6A, P<0.0001) and restored to non-IR levels, indicating that *ztf-8* mutants are experiencing DNA damage-induced apoptosis. Taken together, these studies indicate a function for ZTF-8 in the 9-1-1 mediated meiotic DNA damage checkpoint.

### ZTF-8 is not required for regulating either meiotic crossover frequency or distribution

**Figure 7:**
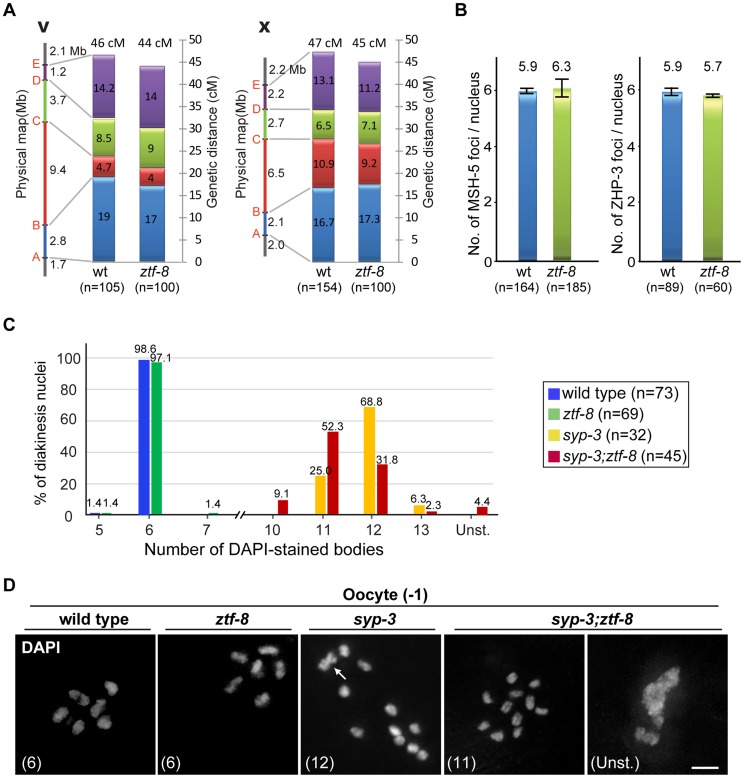
ZTF-8 is not required for the regulation of either crossover frequency or distribution. **A**. Analysis of crossover frequency and distribution for chromosomes V and X in wild type and *ztf-8* mutants. Positions of SNP markers delimiting four intervals (A–B, B–C, C–D, and D–E) are indicated. n = number of cross progeny scored. **B**. Quantitation of the number of MSH-5 and ZHP-3 foci observed in meiotic nuclei from *ztf-8* mutants compared to wild type. MSH-5 foci, P = 0.2139, n = 164 for wt and n = 185 for *ztf-8*. ZHP-3 foci, P = 0.2505, n = 89 for wt and n = 60 for *ztf-8*. **C**. Number of DAPI-stained bodies observed in diakinesis oocytes from the indicated genotypes. The number of −1 oocytes scored (n) is indicated next to the genotypes. Unst., nuclei with unstructured chromatin. **D**. Representative images of DAPI-stained nuclei in −1 oocytes at diakinesis from the indicated genotypes. The number in parenthesis in each image represents the total number of DAPI-stained bodies scored for that nucleus. Unst., nuclei with unstructured chromatin. Arrow indicates two superimposed univalents in the *syp-3(ok758)* diakinesis oocyte.

The increased levels of RAD-51 foci observed in mid to late pachytene suggest a role for ZTF-8 in DSBR via homologous recombination during meiosis. To determine whether ZTF-8 plays a role in meiotic crossover formation we examined crossover frequency and distribution in both an autosome (V) and a sex chromosome (X) in *ztf-8* mutants compared with wild type (Figure 7A). 46.6 cM and 47 cM intervals, corresponding to 81% and 76% of the whole length (interval A to E) of chromosomes V and X, were examined utilizing 5 single-nucleotide polymorphism (SNP) markers along each chromosome as in [18]. Crossover frequency in this interval was weakly, but not significantly, reduced by 5.5% on chromosome V and 4.1% on the X chromosome compared to wild type (P = 0.7657 and P = 0.8872, respectively, by the two-tailed Fisher's exact test, C.I. 95%). Furthermore, the crossover distribution patterns were not altered in either the autosome or the sex chromosome. Crossover distribution was still biased to the terminal thirds of autosomes and somewhat evenly distributed along the X chromosome as demonstrated in [23]. These results suggest that ZTF-8 is not required for the regulation of either crossover frequency or distribution in the autosomes and the sex chromosome.

Further evidence indicates that ZTF-8 does not affect crossover formation. First, the levels of ZHP-3 and MSH-5 foci, which are proposed to mark crossover sites, were indistinguishable between wild type and *ztf-8* mutants (Figure 7B; [24]–[26]. Second, mostly six pairs of attached homologous chromosomes, at levels similar to wild type, were detected in late diakinesis oocytes in *ztf-8* mutants, suggesting that crossover formation resulted in the formation of functional chiasmata (Figure 7C). Therefore, these data indicate that while ZTF-8 is required for normal DSB repair progression it is not required for completion of interhomolog crossover formation.

### ZTF-8 can contribute to intersister repair in the absence of interhomolog crossovers

Both *brc-1* and *fcd-2* mutants exhibit accumulation of RAD-51 foci but normal levels of crossovers, and are required for meiotic DSB repair using sister chromatids when homologous chromosomes are not available [27], [28]. To test if ZTF-8 is required for intersister repair, we employed a *syp-3(ok758)* null mutant background in which meiotic DSB formation still takes place but chromosomes no longer synapse and therefore interhomolog recombination is abrogated due to the lack of a stably held homologous chromosome that can be utilized as a template for repair (Figure 7D; [29]). While we did not observe any evidence of chromosome fragmentation, we found that 4.4% of oocytes at diakinesis exhibited misshapen, unstructured chromatin in the double mutants but not in the *syp-3* mutants (0/32 in *syp-3* and 2/45 in *syp-3;ztf-8*). Similar unstructured chromatin was observed in *brc-1*;*syp-2* mutants, also impaired for chromosome synapsis, albeit at an approximately 6-fold higher frequency [27], suggesting only a modest contribution by ZTF-8 to intersister repair when interhomolog repair is abrogated during meiosis.

### The localization of ZTF-8 is altered in response to DNA damage and requires the ATL-1 and ATM-1 protein kinases

To determine whether ZTF-8 localization might be altered in response to either replication arrest or DSBs we exposed wild type worms to either HU or γ-IR, respectively, and monitored ZTF-8 localization in the germline. Unlike the unexposed germlines, in which ZTF-8 signal is present in nuclei at the premeiotic tip and in late pachytene and only very weak signal is observed at transition zone (Figure 2A), brighter ZTF-8 foci were observed from premeiotic tip to transition zone with HU treatment (Figure 6C). ZTF-8 also formed bright aggregates or foci following γ-IR treatment in nuclei from the premeiotic tip to the pachytene stage. These bright foci started to appear 15 minutes after irradiation, increased in intensity at 30 minutes and started to disappear 120 minutes after irradiation, suggesting a transient nature to this change in localization (Figure 6C and Figure S5). While most of the large foci apparent after either γ-IR or HU treatment were localized to the nucleolus, some were also present associated with chromatin. Specifically, 19% (n = 45 nuclei) of the large foci were associated with the DAPI signal in premeiotic tip nuclei following HU treatment, and 26% (n = 115) and 21% (n = 75) in premeiotic tip and pachytene nuclei, respectively, following γ-IR, while these large foci were rarely observed at either stage in untreated worms. Given the higher levels of smaller foci observed associated with chromatin in non-IR worms, these results suggest that ZTF-8 may be relocalizing after exogenous DNA damage, becoming enriched at or near sites of damage. Consistent with our assessment for specificity in DNA damage sensitivity (Figure 4A) we did not observe altered localization of ZTF-8 following exposure to either HN2, UV or CPT (Figure S6) suggesting a specific response primarily to replication arrest and DSB formation.

Interestingly, both ATL-1 and ATM-1 (ATM homolog) are required for the proper localization of ZTF-8. Specifically, ZTF-8 is observed forming larger foci in premeiotic tip nuclei in both *atl-1* and *atm-1* mutants compared to wild type (Figure 6D). However, at the pachytene stage, where crossover recombination is completed, these larger ZTF-8 foci were only observed in *atl-1* mutants, but not *atm-1*, suggesting that ZTF-8 localization is dependent on ATL-1 during both mitotic and meiotic progression. Consistent with this idea, ZTF-8 acquires the same enlarged focal pattern in *atm-1;atl-1* double mutants as that observed throughout the germlines of *atl-1* single mutants. These observations suggest that proper localization of ZTF-8 requires both ATL-1 and ATM-1 during mitosis and early meiosis, but mostly only ATL-1 during late meiotic prophase. However, given the pleiotropic nature of the *atm-1* and *atl-1* mutants, we cannot exclude the possibility that the localization of ZTF-8 is altered as a result of alterations to the pattern of damaged DNA.

### ZTF-8 partially co-localizes with HUS-1, a member of the 9-1-1 complex, and both ZTF-8 and HUS-1 are interdependent for their nuclear localization

To further examine the nature of the large ZTF-8 foci observed in response to DNA damage, we assessed whether ZTF-8 co-localizes with any known DDR or DNA repair proteins at either 10 or 30 minutes post γ-IR treatment, compared to untreated gonads. We failed to detect co-localization between ZTF-8 and MRE-11 (involved in DSB resection; [30], [31], RPA-1 (single-stranded DNA binding protein; [32]), RAD-51 (strand invasion/exchange protein; [33]) and RAD-54 (required for removal of RAD-51; [34]). However, utilizing a HUS-1::GFP transgenic line we found partial co-localization between ZTF-8 and HUS-1 predominantly in the absence of γ-IR exposure (Figure 6E). Specifically, 82% of HUS-1::GFP foci co-localized with ZTF-8 foci at the premeiotic tip (n = 62 nuclei from 5 germlines). However this level decreased to 9% after γ-IR treatment (n = 46 nuclei from 4 germlines). We observed a similar trend in pachytene nuclei. However, the presence of various stretches and clusters of foci for HUS-1::GFP precluded us from quantifying the degree of co-localization at this stage. Interestingly, 67% of the foci observed co-localizing at the premeiotic tip did not localize to DAPI-stained chromosomes, suggesting that their co-localization may be taking place outside of repair foci when exogenous DSBs are absent. Of note, the HUS-1::GFP transgene has been reported to only partially rescue the apoptotic defect observed in *hus-1(op241)* mutants [8], and the HUS-1::GFP signal is weak, especially in the absence of exogenous DSBs, the level of co-localization observed between HUS-1::GFP and ZTF-8 might represent an underestimate. Finally, ZTF-8 signal was reduced in *hus-1* mutants compared to wild type at both the premeiotic and pachytene stages (Figure 6F), complementing our observation of a decrease in HUS-1 signal in *ztf-8* mutants (Figure 6B). Altogether, these data indicate that HUS-1 and ZTF-8 are partly interdependent for their localization and suggest a potential, either direct or indirect, interaction between these proteins.

### ZTF-8 interacts with MRT-2, a member of the 9-1-1 complex, and is the functional ortholog of human RHINO

**Figure 8:**
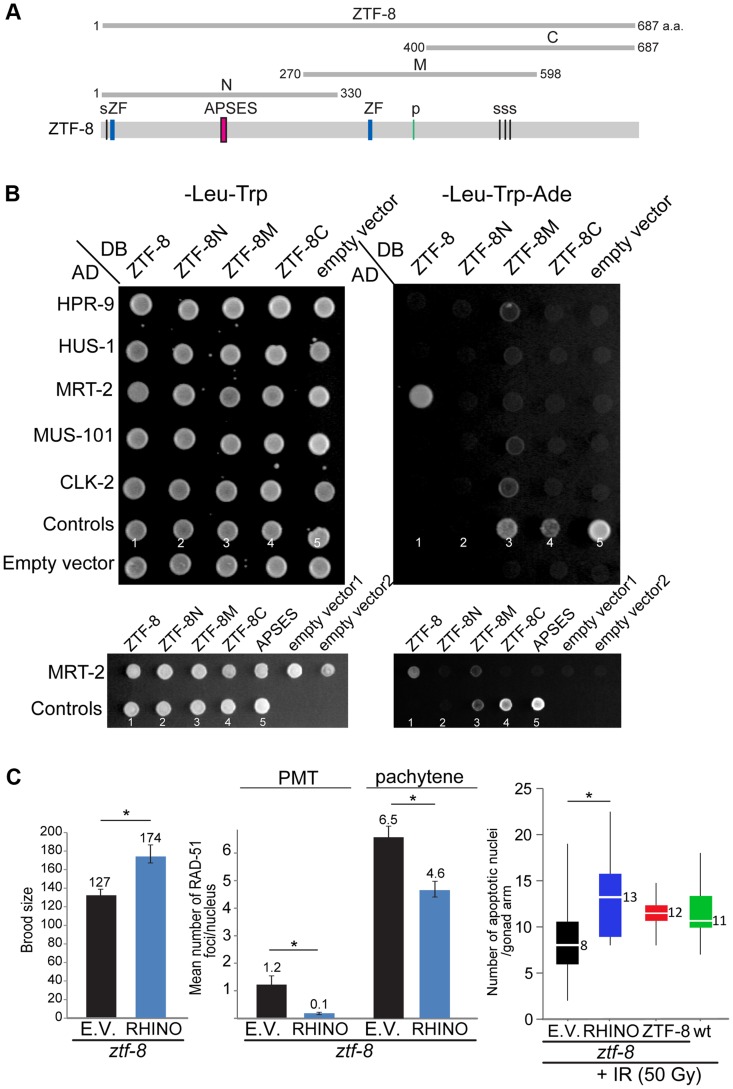
ZTF-8 interacts with MRT-2/Rad1 and shares functional conservation with mammalian RHINO. **A**. Schematic representation of the region of ZTF-8 used in the yeast two-hybrid assay. **B**. The yeast two-hybrid system was used to test the protein interactions between ZTF-8, HPR-9, HUS-1, MRT-2, MUS-101 and CLK-2. Both full length and truncations of ZTF-8 were examined. Only full length ZTF-8 interacts with MRT-2. A mutation (SSLCPNA to AAAAAAA) at the predicted DNA binding site (APSES) in the N-terminal region of ZTF-8 abrogates its binding interaction to MRT-2. Proteins were fused to either the DNA binding domain (DB) or the activation domain (AD) of GAL4. Interactions were scored by growth on SC-Leu-Trp-Ade plates. One negative (No. 1) and four positive controls (No. 2-5) were used as described in [64]. Control No. 1: pPC97(DB) and pPC86(AD) is a negative control; control No. 2: pPC97-RB(DB) and pPC86-E2F1(AD) is a weak interaction; control No. 3: pPC97-CYH2s-dDP(DB) and pPC86-dE2F(AD) is a moderate interaction; control No. 4: pPC97-FOS(DB) and pPC86-JUN(AD) is a strong interaction; control No. 5: pCL-1(GAL4)(DB) and pPC86(AD) is a very strong interaction. **C**. Expression of mammalian RHINO rescues the decreased brood size, elevated levels of RAD-51 foci and reduced level of apoptosis observed in *ztf-8* mutants. *ztf-8* mutant worms expressing RHINO are compared to *ztf-8* mutants carrying either an empty vector (E.V.) or wild type ZTF-8 as a control. Brood size, P = 0.0128 by the two-tailed Mann-Whitney test, 95% C.I. n = 13 worms for control and n = 5 for RHINO. Levels of RAD-51 foci were scored in nuclei at the premeiotic tip (PMT, zone1) and in pachytene (zone 5), P<0.0001 for both PMT and pachytene regions by the two-tailed Mann-Whitney test, 95% C.I. In the PMT, n = 171 (control) and n = 249 (RHINO). In pachytene, n = 81(control) and n = 127 (RHINO). Levels of apoptosis are measured 20 hours after exposure to γ-IR (50 Gy). Asterisk indicates statistical significance. P = 0.0004, n = 22 gonads for empty vector, n = 20 for RHINO, n = 16 for ZTF-8 and n = 20 for wild type.

To examine whether ZTF-8 interacts with any of the members of the 9-1-1 complex we applied a yeast two-hybrid approach. We tested the full length and three specific regions of ZTF-8 (N^1–330^, M^270–598^ and C^400–687^) for interactions with potential candidates (Figure 8A). ZTF-8N includes a putative sumoylation site, a zinc-finger domain and the predicted APSES DNA binding motif. ZTF-8M contains a zinc-finger domain and a putative phosphorylation site. ZTF-8C contains three putative sumoylation sites. Interestingly, an interaction was observed between MRT-2/Rad1, and the full length ZTF-8 (Figure 8B). A lack of detectable interaction between MRT-2 and any of the ZTF-8 truncations suggests that the N, M, and C regions alone may not be sufficient to sustain an interaction with MRT-2.

Similar to the human RHINO protein [13], a mutation in the conserved APSES DNA binding domain (SSLCPNA to AAAAAAA) abolished the binding affinity to MRT-2 suggesting that the APSES domain is required for the interaction between the member of the 9-1-1 complex and ZTF-8 (Figure 8B and Figure S1). The human RHINO protein was shown to co-immunoprecipitate with TopBP1 and Rad9 suggesting a link to the 9-1-1 complex [13], although a direct protein interaction with any of the 9-1-1 complex members was not demonstrated. Full-length ZTF-8 does not interact via a yeast two-hybrid method with HPR-9 (Rad9 homolog), MUS-101 (TopBP1 DNA topoisomerase 2 beta binding protein), and HUS-1, suggesting that the connection with the 9-1-1 complex may be via MRT-2. CLK-2 (*S. cerevisiae* Tel2p ortholog), which was previously reported to exhibit synthetic sterility with ZTF-8 [35], also did not interact with the full-length ZTF-8 by this assay. Importantly, similar results were obtained using different combinations of yeast strains and plasmids, further supporting these observed interactions. However, a mild interaction was observed between CLK-2 and the ZTF-8M truncation. Given that only this ZTF-8 truncation also exhibits mild interactions with HPR-9 and MUS-101, these may be false positive (non-specific) interactions resulting from either the misfolding of this truncated protein or it being “sticky”.

To examine if ZTF-8 and RHINO indeed share functional conservation, transgenic lines expressing RHINO were tested for their ability to rescue the phenotypes observed in *ztf-8* mutant animals. Human RHINO rescued the reduced brood size, elevated levels of RAD-51 foci and impaired germ cell apoptosis observed in *ztf-8* mutants (Figure 8C). Altogether, these data support a role for ZTF-8, the functional RHINO homolog, in promoting the proper activation of the DNA damage checkpoint by interacting with MRT-2/Rad1 a component of the 9-1-1 complex. Our studies also suggest that RHINO may be directly connected to the 9-1-1 complex in a similar manner and that it may play a role in maintaining genomic integrity during meiosis in humans.

## Discussion

**Figure 9:**
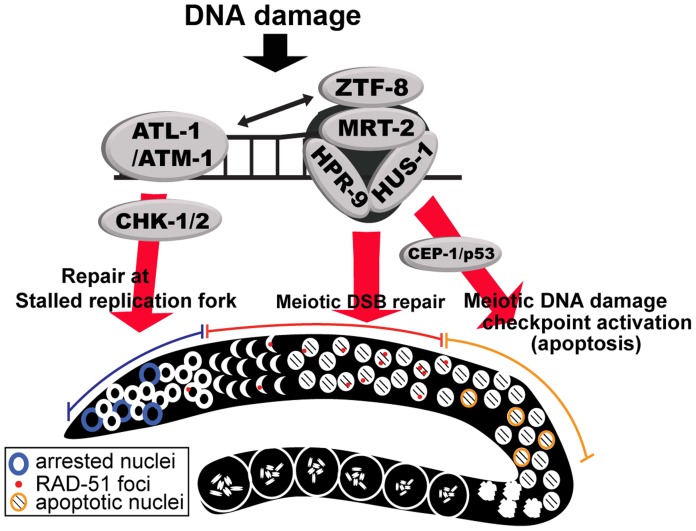
Model for the role of ZTF-8 in DNA damage response and repair. DNA damage induces DNA replication arrest and DSB repair in the germline. First, stalled replication forks in mitotic dividing cells induce the S-phase cell cycle checkpoint via activation of the ATL-1- and CHK-1-dependent pathway. Second, programmed meiotic DSBs will be repaired by homologous recombination, whereas persistent unrepaired DSBs and/or aberrant recombination intermediates will be removed by apoptosis via activation of the CEP-1/p53-mediated DNA damage checkpoint. ZTF-8 participates both in the activation of the DNA damage checkpoint and in DSBR. We propose that ZTF-8 acts via the 9-1-1 complex in transducing DNA damage signaling for repair of both mitotic and meiotic DSBs and meiotic germ cell apoptosis.

Impaired *ztf-8* function results in a reduced brood size, mild embryonic lethality and increased levels of X-chromosome non-disjunction. The hypersensitivity of *ztf-8* mutants to exogenous DSBs and replication arrest, coupled with the increased levels of recombination intermediates detected in both mitotic and meiotic germline regions, and the presence of chromatin fragments marked by RAD-51 foci, all strongly support a role for ZTF-8 in homologous recombination repair in *C. elegans*. In addition to its role in DSBR, our studies suggest that ZTF-8 acts in the DNA damage checkpoint pathway, consistent with the role of RHINO in human cells. This idea is further supported by the fact that proper localization of ZTF-8 requires both ATL-1 and ATM-1, which are kinases central for DNA-damage response [36]. ZTF-8 might be a direct target for phosphorylation by ATM/ATR given that S/TQ sites, shown to undergo such phosphorylation following DNA damage, are present in ZTF-8 (Figure S1) [37]. Furthermore, both the observed synthetic lethality with *clk-2* and decreased HUS-1::GFP signal in *ztf-8* mutants strongly implicates *ztf-8* in DNA damage response. We demonstrated that ZTF-8 partially co-localizes with the 9-1-1 complex and interacts with MRT-2 in a manner dependent on the presence of the APSES domain. We propose that ZTF-8 is involved in promoting repair at stalled replication forks and meiotic DSBs in part by transducing DNA damage checkpoint signaling via the 9-1-1 pathway (Figure 9).

### ZTF-8 is required for repair of stalled replication forks and programmed meiotic DSBs

Increased levels of RAD-51 foci were observed in both mitotic and meiotic zones in the germlines of the *ztf-8* mutants (Figure 4D). What causes accumulation of RAD-51 foci in *ztf-8* mutants? The activation of the S-phase cell cycle arrest in *ztf-8* mutants indicates that the mitotic increase in the levels of RAD-51 foci likely stems from a role for ZTF-8 in repair at stalled or collapsed replication forks. This idea is supported by the increased nuclear diameter and HU induced embryonic and larval lethality observed in the mutants (Figure 3A and 4A). On the other hand, the elevated levels of RAD-51 foci during meiosis can be explained by the progression of unrepaired breaks of mitotic origin to the meiotic stages as well as defective DSBR during meiosis *per se*, as evidenced by comparing the levels of mitotic to meiotic (SPO-11-dependent) DSBs (Figure 4D).

How does ZTF-8 work in the repair at stalled or collapsed replication forks and SPO-11-induced DSBs? We considered the possibility that ZTF-8 functions as a part of the Shu complex, which has been reported to suppress the HU sensitivity observed in mutants of SGS1, which encodes the budding yeast homolog of the BLM helicase and, similar to ZTF-8, is primarily localized to the nucleolus. However, unlike *ztf-8* mutants where unrepaired DSBs persist, the number of RAD51 foci in a *shu1* deletion strain is decreased compared to wild type [38]. Moreover, no important amino acid conservation is found between ZTF-8 and the Shu components or their human homologs [38], [39]. Given that ZTF-8 is largely localized to the nucleolus in nuclei at the mitotic zone, we examined whether it might play a role in maintaining G/C tracts, which have the potential to adopt secondary structures such as the G-quadruplex and thus induce DNA replication arrest. However, we did not detect significant changes in the sizes of the GC tracts found in either *ztf-8* single or *dog-1;ztf-8* double mutants (n = 41 for each), where DOG-1 is the *C. elegans* homolog of the FANCJ helicase previously implicated in poly(G)/poly(C) (G/C) tract maintenance during DNA replication [40], [41].

Recent studies found that the RNA:DNA hybrid structures known as R-loops formed between nascent mRNA and template DNA during transcription can impair replication and cause checkpoint activation during meiosis, mimicking similar phenotypes found in *ztf-8* mutants and suggesting a possible involvement of ZTF-8 in antagonizing R-loop formation [42], [43]. Alternatively, ZTF-8 might be required for the translesion synthesis (TLS) pathway given that it carries a potential ubiquitin-binding zinc-finger (UBZ) domain found in TLS polymerases and that defective TLS repair results in accumulation of RAD-51 foci [44]. Although *ztf-8* mutants are not sensitive to DNA interstrand crosslinks (Figure 4A), a role in the TLS pathway is still plausible as not all UBZ-containing TLS components, such as POLK-1/POLκ, are sensitive to ICLs [45]. This idea is further supported by observations that the 9-1-1 complex is required for recruiting translesion polymerases to stalled replication forks in *S. pombe* [46], [47], and therefore the absence of ZTF-8 might lead to the accumulation of unrepaired breaks. In fact, the two observed functions for ZTF-8 in DNA repair and DNA damage checkpoint activation correspond to the two distinct roles ascribed to the 9-1-1 complex: 1) checkpoint signaling through ATM and ATR to stimulate the DNA repair pathway; and 2) as a recruitment platform for the TLS machinery at stalled replication forks [48], [49]. Our results suggest that ZTF-8 is required for both functions of 9-1-1 and we hypothesize that the former might be its prevalent mode of action during meiosis and the latter its primary mode of action during S-phase.

### ZTF-8 is required for 9-1-1 mediated DNA damage response signaling

Late pachytene nuclei carrying unrepaired DSBs, as visualized by RAD-51 immunostaining in *ztf-8* mutants, can activate a DNA damage checkpoint at that stage and be converted into apoptotic nuclei [18], [22]. Accumulation of ATL-1 further supports the activation of the DNA damage checkpoint via the CEP-1/p53 pathway in the *ztf-8* mutants [16]. However, levels of germ cell apoptosis following the induction of exogenous DSBs were not as elevated as in wild type. A simple explanation is that ZTF-8 is required for proper function of either the apoptotic machinery or the DNA damage mediated apoptosis pathway. However, normal levels of physiological germ cell apoptosis are still present in the *ztf-8* mutants, suggesting that ZTF-8 is not required for the apoptotic machinery (Figure 6A). Moreover, in *cep-1;ztf-8* double mutants the level of apoptosis was comparable to that of *cep-1* mutants supporting an impaired DNA damage checkpoint in *ztf-8* mutants.

In the *C. elegans* germline HUS-1 and CEP-1/p53 act in the same pathway and HUS-1 is required for the CEP-1/p53-dependent DNA damage induced apoptosis [8]. Our observations of a weaker HUS-1::GFP signal in *ztf-8* mutants either in the presence or in the absence of exogenous DSBs, the interaction between ZTF-8 and the 9-1-1 complex via MRT-2, and the weak levels of apoptosis despite the elevated levels of unrepaired recombination intermediates highlighted by RAD-51 foci present in late pachytene, suggest that ZTF-8 is required for the intact DNA damage response signaling pathway.

The kinetics of HUS-1::GFP localization are different from that of ZTF-8. ZTF-8 partially co-localizes with HUS-1::GFP, a component of the 9-1-1 DNA damage checkpoint, both in the nucleolus and on chromatin at mitotic and meiotic stages when no exogenous DSBs are present. ZTF-8 starts to form bright foci as early as 15 min after γ-IR treatment, but the number of bright foci starts to decrease 2 hr after irradiation while HUS-1::GFP not yet forms distinct foci at chromatin. Importantly, ZTF-8 does not co-localize with the HUS-1::GFP bright and distinct foci that appear on chromatin as early as 3 hr after γ-IR [8]. These observations are consistent with ZTF-8's relocalization after DNA damage and suggest that ZTF-8 is required for proper 9-1-1-mediated signaling, co-localizing with the complex until DSBs occur, upon which the 9-1-1 DNA damage complex re-localizes to DSB sites.

Although ZTF-8 is important for the CEP-1/p53-dependent activation of the meiotic DNA damage checkpoint it is not required for mitotic cell cycle arrest. This is distinct from MRT-2 and HUS-1, which have been previously shown to exhibit both impaired mitotic cell cycle arrest and meiotic DNA damage checkpoint activation [8], [22]. Importantly, mitotic germ cells in *ztf-8* mutants were proficient for G2 arrest following exposure to γ-IR (40 Gy) as observed by a 1.5-fold increase in nuclear diameter that was similar to the 1.4-fold increase observed in IR-treated wild type nuclei compared to the non-IR nuclei (n = 50–138 nuclei each for wt, wt +IR, *ztf-8* and *ztf-8*+IR; P<0.0001 by the two-tailed Mann-Whitney test, 95% C.I.). Therefore, the absence of a detectable mitotic cell cycle arrest defect in *ztf-8* mutants is not simply due to differences in the type of damage induced, namely stalled replication forks in S-phase compared to DSBs in meiotic prophase I. Instead, this further suggests that ZTF-8 may be required for a separate function of the 9-1-1 complex during S-phase, such as possibly in the TLS pathway, and not in checkpoint signaling.

In summary, our study discovered ZTF-8, a previously uncharacterized protein, and its functions in the germline. We have revealed that ZTF-8 plays both mitotic and meiotic roles via its requirements for DSB repair and DNA damage checkpoint activation through an interaction with the 9-1-1 complex. A previous study identified an overexpression of RHINO in breast cancer cells and that its depletion by small-hairpin RNAi suppressed their cell growth [50]. However, no previous studies have been reported on the meiotic roles of RHINO although it is expressed in both testis and ovary in normal human tissues [50]. Here we demonstrated that ZTF-8 and RHINO share functional conservation. Therefore, the insights we provide as to how ZTF-8 is tied into the 9-1-1 complex for repair at stalled replication forks and meiotic DSBs, as well as for the activation of the CEP-1/p53-dependent germ cell apoptosis pathway, shed new light on how RHINO may be operating via the 9-1-1 complex in these different contexts during mammalian mitosis and meiosis.

## Materials and Methods

### Strains and alleles

*C. elegans* strains were cultured at 20°C under standard conditions as described in Brenner [51]. The N2 Bristol strain was used as the wild-type background. The following mutations and chromosome rearrangements were used in this study: LGI: *atm-1(gk186)*, *cep-1(lg12501)*, *hus-1(op244), hT2[bli-4(e937) let-?(q782) qIs48](I; III)*; LGIII: *ztf-8(tm2176), sDf121, qC1[dpy-19(e1259) glp-1(q339) qIs26] (III)*; LGIV: *spo-11(ok79), nT1[unc-?(n754) let-? qIs50](IV; V), nT1[qIs51] (IV; V)*; LGV: *atl-1(tm853)*, *syp-1(me17) [* [52] [53] [51] [54] [55].

The *ztf-8(tm2176)* mutant was generated by the Japanese National BioResource Project for *C. elegans* and carries a 524 base pair out-of-frame deletion that removes most of exon 6 along with exons 7 through 11 (Figure 1). This deletion results in a premature stop codon and loss of the zinc-finger motifs located in the middle of ZTF-8, the four ΨKXE consensus sumoylation sites, and a putative phosphorylation site (http://www.phosphopep.org).

### Transgenic worms

To test if human RHINO can rescue the *ztf-8* mutant phenotypes, RHINO cDNA was cloned into the pID2.02 plasmid, which contains *unc-119(wt)*, then injected into *ztf-8(tm2176)*;*unc-119(ed9)* mutants and screened for wild type (non-Unc) moving worms [56], [57]. Rescued non-Unc worms were irradiated to obtain single/low-copy integration of transgenes as described in [58]. Presence of RHINO was confirmed by PCR. Empty pID2.02 was injected into *ztf-8(tm2176)*;*unc-119(ed9)* as a control.

### Analysis of ZTF-8 protein conservation and motifs

ZTF-8 homology searches and alignments were performed using Uniprot (http://www.uniprot.org/). Pfam and Prosite (release 20.70) were used for zinc-finger motif predictions [59].

### Quantitative analysis for RAD-51 foci

Quantitative analysis of RAD-51 foci was performed as in [20]. Between 5 to 8 germlines were scored for each genotype. The average number of nuclei scored per zone for a given genotype was as follows, ± standard deviation: zone 1, n = 157.4 ±38.3; zone 2, n = 151.8±39.5; zone 3, n = 136.6±27.1, zone 4 = 212.8±69.1, zone 5 = 130.0±49.3, zone 6 = 124.5±56.4, zone 7 = 108.2±46.8. Statistical comparisons between genotypes were performed using the two-tailed Mann-Whitney test, 95% confidence interval (C.I.).

### DNA damage sensitivity experiments

Young adult homozygous *ztf-8* animals were picked from the progeny of *ztf-8*/*qC1* parent animals. To assess for IR sensitivity, animals were treated with 0, 10, 30 or 100 Gy of γ-IR from a Cs^137^ source at a dose rate of 1.8 Gy/min. For HN2 sensitivity, animals were treated with 0, 50, 100 or 150 µM of HN2 (mechlorethamine hydrochloride; Sigma) in M9 buffer containing *E. coli* OP50 with slow shaking in the dark for 20 hours. CPT (Sigma) treatment was similar but with doses of 0, 100, 500, or 1000 nM. After treatment with either HN2 or CPT, animals were washed twice with M9 containing TritonX100 (100 ml/L) and plated to allow recovery for 3 hours [18]. UV irradiation treatment was performed utilizing the XL-100 Spectrolinker UVC. Worms were exposed to 0, 100 or 150 J/m^2^ of UVC and plated to allow recovery for 3 hours. HU sensitivity was assessed by placing animals on seeded NGM plates containing either 0, 10 or 15 mM HU for 20–24 hours. Hatching sensitivity was examined in>24 animals 4 hours after HU treatment. For all other damage sensitivity experiments,>24 animals were plated, 7 per plate, and hatching was assessed for the time period of 20–24 hours following treatment. For L1 genotoxic assays, L1(P0) worms were plated on NGM plates with either 0 or 25 mM HU and incubated for 16 hours. The number of live adult progeny (F1) were counted as described in [19]. Each damage condition was replicated at least twice in independent experiments.

### RNA interference

Feeding RNAi experiments were performed at either 20°C or 25°C as described in [60]. Either the entire coding sequence of *ztf-8* (Geneservice) or cDNA corresponding to its C-terminal 501 bp cloned into the pL4440 feeding vector were used for RNAi experiments. HT115 bacteria carrying the empty pL4440 vector were used as control RNAi.

cDNA was produced from single-worm RNA extracts using the One step RT-PCR system (USB). The effectiveness of RNAi was examined by assaying the expression of the transcript being depleted in four individual animals subjected to RNAi by feeding. Expression of the *myo-3* (K12F2.1) transcript was used as a control.

### Antibody production and immunofluorescence

Rabbit polyclonal antibodies against N- and C-terminal peptides of *C. elegans* ZTF-8 (ETLKEEGAHFYKHFKYKRYC and CHHSRSSYRGNRDDRGSRW, respectively) were generated by Yenzym antibodies, LLC. Antisera were affinity-purified using SulfoLink (Pierce) following the manufacturer's instructions.

Whole mount preparations of dissected gonads, fixation and immunostaining procedures were carried out as described in [20]. Primary antibodies were used at the following dilutions: rabbit α-ZTF-8 (1∶200), rabbit α-ATL-1 (1∶500; [16]), rabbit α-RAD-51 (1∶2000; SDIX), mouse α-NOP-1 (1∶100; EnCor Biotech), rabbit α-PCN-1 (1∶10000; [61]), rabbit α-phospho Ser10 Histone H3 (1∶200; Upstate Biotechnologies), guinea pig α-SYP-1 (1∶200; [62]), rabbit α-SMC-3(1∶100; Chemicon), rabbit α-HDA-1 (1∶200; Santa Cruz), rabbit α-Histone H3 (1∶200; Cell Signaling), rabbit α-CDK1 pTyr15 (1∶50; Calbiochem) and rabbit α-pCHK-1 (1∶50; Santa Cruz). Secondary antibodies used were: Cy3 anti-rabbit, FITC anti-rabbit, Cy3 anti-guinea pig and FITC anti-mouse (Jackson Immunochemicals), each at 1∶200.

Immunofluorescence images were collected at 0.2 µm intervals with an IX-70 microscope (Olympus) and a CoolSNAP HQ CCD camera (Roper Scientific) controlled by the DeltaVision system (Applied Precision). Images were subjected to deconvolution by using the SoftWoRx 3.3.6 software (Applied Precision).

### Quantitative analysis of germ cell apoptosis

Germlines of age-matched (20 hours post-L4) animals were analyzed by acridine orange staining, as described in [24], utilizing a Leica DM5000B fluorescence microscope. Between 22 and 95 gonads were scored for each genotype. Statistical comparisons between genotypes were performed using the two-tailed Mann-Whitney test, 95% C.I.

### Yeast two-hybrid screen

The full-length of the *ztf-8* open reading frame, as well as C- (400–687), middle (270–598) and N- (1–330) terminal truncations were amplified by PCR. A cDNA library generated from mixed-stage *C. elegans* was used for the amplification with primers that contain Gateway compatible sequences and a gene specific sequence as indicated in Table S1. Gateway cloning, cDNA and ORFeome library screening, and X-Gal, -URA and 3AT assays for examining yeast two hybrid interactions were performed as in [63].

## Supporting Information

Figure S1 ZTF-8 protein conservation. Sequence alignment between *C. elegans* ZTF-8 and its predicted homologs in *H. sapiens, M. musculus, X. tropicalis, N. vectensis, S. Salar*, and *T. porphyreolophus*. Alignment was performed using CLUSTAL 2.1 from EMBL-EBI (www.ebi.ac.uk) and Pfam (http://pfam.sanger.ac.uk). Shaded dark blue boxes indicate amino acid identity and light blue boxes indicate similarity. 8% identity and 15% of amino acid sequence similarity was found between RHINO (*H. sapiens*) and ZTF-8 (*C. elegans*) by using CLUSTAL 2.1. Zinc-finger motifs were identified using Prosite (http://prosite.expasy.org) and are underlined with red lines. A red-colored box indicates the hypothetical APSES DNA binding site found in different species. Black-colored boxes indicate SQ and TQ sites.
(TIF)

Figure S2 ZTF-8 localization to somatic nuclei. Co-staining of intestinal nuclei from wild type and *ztf-8* mutants with DAPI (blue), an anti-NOP-1 antibody (red) and an anti-ZTF-8 antibody (green). Bar, 2 µm.
(TIF)

Figure S3 Assessing mitotic progression by immunostaining with G2/M markers. Immunostaining of wild type and *ztf-8* mutants with DAPI and an anti CDK1 pTyr15 antibody or an anti phospho-histone H3 pSer10 antibody. *ztf-8* mutants exhibit similar staining pattern in either CDK-1 (n = 8 germlines for both genotypes) or pH 3 staining (n = 18 for wild type and n = 19 for *ztf-8* mutants, P = 0.9139 by the two-tailed Mann-Whitney test, 95% C.I.). Bar, 2 µm.
(TIF)

Figure S4 Assessing DSBR progression by quantitation of RAD-51 foci. Graphs depict the percentage of nuclei carrying RAD-51 foci (y-axis) within each zone along the germline (x-axis). Asterisks indicate statistical significance compared to either wild type (*) or *spo-11* (*).
(TIF)

Figure S5 ZTF-8 localization changes in response to exogenous DSB formation. Immunolocalization of ZTF-8 prior to and 15, 30 and 120 minutes following γ-IR exposure (50 Gy). PMT, premeiotic tip; TZ, transition zone. Bar, 2 µm.
(TIF)

Figure S6 ZTF-8 localization does not change in response to HN2, UV and CPT treatment. Immunolocalization of ZTF-8 30 minutes after exposure to UVC (150 J/m2), CPT (500 nM), and HN2 (150 µM). Control contains DMSO only. Bar, 2 µm.
(TIF)

Table S1 Primers used for the yeast two-hybrid experiments. These primers were utilized to generate the full length and truncations of ZTF-8.
(DOC)

## References

[1] Lengauer C, Kinzler KW, Vogelstein B (1998) Genetic instabilities in human cancers. Nature 396: 643–649. 9872311 10.1038/25292

[2] Kennedy RD, D'Andrea AD (2006) DNA repair pathways in clinical practice: lessons from pediatric cancer susceptibility syndromes. J Clin Oncol 24: 3799–3808. 16896009 10.1200/JCO.2005.05.4171

[3] Bachrati CZ, Hickson ID (2003) RecQ helicases: suppressors of tumorigenesis and premature aging. Biochem J 374: 577–606. 12803543 10.1042/BJ20030491 PMC1223634

[4] Lynch HT, de la Chapelle A (1999) Genetic susceptibility to non-polyposis colorectal cancer. J Med Genet 36: 801–818. 10544223 PMC1734258

[5] Vasen HF, Watson P, Mecklin JP, Lynch HT (1999) New clinical criteria for hereditary nonpolyposis colorectal cancer (HNPCC, Lynch syndrome) proposed by the International Collaborative group on HNPCC. Gastroenterology 116: 1453–1456. 10348829 10.1016/s0016-5085(99)70510-x

[6] Murakami H, Nurse P (2000) DNA replication and damage checkpoints and meiotic cell cycle controls in the fission and budding yeasts. Biochem J 349: 1–12. 10861204 10.1042/0264-6021:3490001 PMC1221113

[7] Guo Z, Wang J, Meng L, Wu Q, Kim O, et al (2001) Cutting edge: membrane lymphotoxin regulates CD8(+) T cell-mediated intestinal allograft rejection. J Immunol 167: 4796–4800. 11673481 10.4049/jimmunol.167.9.4796

[8] Hofmann ER, Milstein S, Boulton SJ, Ye M, Hofmann JJ, et al (2002) Caenorhabditis elegans HUS-1 is a DNA damage checkpoint protein required for genome stability and EGL-1-mediated apoptosis. Curr Biol 12: 1908–1918. 12445383 10.1016/s0960-9822(02)01262-9

[9] Stergiou L, Hengartner MO (2004) Death and more: DNA damage response pathways in the nematode C. elegans. Cell Death Differ 11: 21–28. 14685168 10.1038/sj.cdd.4401340

[10] Craig AL, Moser SC, Bailly AP, Gartner A (2012) Methods for studying the DNA damage response in the Caenorhabdatis elegans germ line. Methods Cell Biol 107: 321–352. 22226529 10.1016/B978-0-12-394620-1.00011-4

[11] O'Neil NJ, Rose AM (2006) DNA repair. Wormbook 13: 11–12. 10.1895/wormbook.1.54.1 PMC4781471 18050489

[12] Colaiacovo MP, Stanfield GM, Reddy KC, Reinke V, Kim SK, et al (2002) A targeted RNAi screen for genes involved in chromosome morphogenesis and nuclear organization in the Caenorhabditis elegans germline. Genetics 162: 113–128. 12242227 10.1093/genetics/162.1.113 PMC1462232

[13] Cotta-Ramusino C, McDonald ER 3rd, Hurov K, Sowa ME, Harper JW, et al (2011) A DNA damage response screen identifies RHINO, a 9-1-1 and TopBP1 interacting protein required for ATR signaling. Science 332: 1313–1317. 21659603 10.1126/science.1203430 PMC4357496

[14] Iyer LM, Koonin EV, Aravind L (2002) Extensive domain shuffling in transcription regulators of DNA viruses and implications for the origin of fungal APSES transcription factors. Genome Biol 3: RESEARCH0012. 11897024 10.1186/gb-2002-3-3-research0012 PMC88810

[15] Bartek J, Lukas C, Lukas J (2004) Checking on DNA damage in S phase. Nat Rev Mol Cell Biol 5: 792–804. 15459660 10.1038/nrm1493

[16] Garcia-Muse T, Boulton SJ (2005) Distinct modes of ATR activation after replication stress and DNA double-strand breaks in Caenorhabditis elegans. EMBO J 24: 4345–4355. 16319925 10.1038/sj.emboj.7600896 PMC1356337

[17] Karnani N, Dutta A (2011) The effect of the intra-S-phase checkpoint on origins of replication in human cells. Genes Dev 25: 621–633. 21406556 10.1101/gad.2029711 PMC3059835

[18] Saito TT, Youds JL, Boulton SJ, Colaiacovo MP (2009) Caenorhabditis elegans HIM-18/SLX-4 interacts with SLX-1 and XPF-1 and maintains genomic integrity in the germline by processing recombination intermediates. PLoS Genet 5: e1000735. 19936019 10.1371/journal.pgen.1000735 PMC2770170

[19] Bailly AP, Freeman A, Hall J, Declais AC, Alpi A, et al (2010) The Caenorhabditis elegans homolog of Gen1/Yen1 resolvases links DNA damage signaling to DNA double-strand break repair. PLoS Genet 6: e1001025. 20661466 10.1371/journal.pgen.1001025 PMC2908289

[20] Colaiacovo MP, MacQueen AJ, Martinez-Perez E, McDonald K, Adamo A, et al (2003) Synaptonemal complex assembly in C. elegans is dispensable for loading strand-exchange proteins but critical for proper completion of recombination. Dev Cell 5: 463–474. 12967565 10.1016/s1534-5807(03)00232-6

[21] Gasior SL, Olivares H, Ear U, Hari DM, Weichselbaum R, et al (2001) Assembly of RecA-like recombinases: distinct roles for mediator proteins in mitosis and meiosis. Proc Natl Acad Sci U S A 98: 8411–8418. 11459983 10.1073/pnas.121046198 PMC37451

[22] Gartner A, Milstein S, Ahmed S, Hodgkin J, Hengartner MO (2000) A conserved checkpoint pathway mediates DNA damage–induced apoptosis and cell cycle arrest in C. elegans. Mol Cell 5: 435–443. 10882129 10.1016/s1097-2765(00)80438-4

[23] Barnes TM, Kohara Y, Coulson A, Hekimi S (1995) Meiotic recombination, noncoding DNA and genomic organization in Caenorhabditis elegans. Genetics 141: 159–179. 8536965 10.1093/genetics/141.1.159 PMC1206715

[24] Kelly KO, Dernburg AF, Stanfield GM, Villeneuve AM (2000) Caenorhabditis elegans msh-5 is required for both normal and radiation-induced meiotic crossing over but not for completion of meiosis. Genetics 156: 617–630. 11014811 10.1093/genetics/156.2.617 PMC1461284

[25] Tzur YB, Egydio de Carvalho C, Nadarajan S, Van Bostelen I, Gu Y, et al (2012) LAB-1 targets PP1 and restricts Aurora B kinase upon entrance into meiosis to promote sister chromatid cohesion. PLoS Biol 10: e1001378. 22927794 10.1371/journal.pbio.1001378 PMC3424243

[26] Bhalla N, Wynne DJ, Jantsch V, Dernburg AF (2008) ZHP-3 acts at crossovers to couple meiotic recombination with synaptonemal complex disassembly and bivalent formation in C. elegans. PLoS Genet 4: e1000235. 18949042 10.1371/journal.pgen.1000235 PMC2567099

[27] Adamo A, Montemauri P, Silva N, Ward JD, Boulton SJ, et al (2008) BRC-1 acts in the inter-sister pathway of meiotic double-strand break repair. EMBO Rep 9: 287–292. 18219312 10.1038/sj.embor.7401167 PMC2267377

[28] Adamo A, Collis SJ, Adelman CA, Silva N, Horejsi Z, et al (2010) Preventing nonhomologous end joining suppresses DNA repair defects of Fanconi anemia. Mol Cell 39: 25–35. 20598602 10.1016/j.molcel.2010.06.026

[29] Smolikov S, Eizinger A, Schild-Prufert K, Hurlburt A, McDonald K, et al (2007) SYP-3 restricts synaptonemal complex assembly to bridge paired chromosome axes during meiosis in Caenorhabditis elegans. Genetics 176: 2015–2025. 17565948 10.1534/genetics.107.072413 PMC1950610

[30] Symington LS, Gautier J (2011) Double-strand break end resection and repair pathway choice. Annu Rev Genet 45: 247–271. 21910633 10.1146/annurev-genet-110410-132435

[31] Stracker TH, Petrini JH (2011) The MRE11 complex: starting from the ends. Nat Rev Mol Cell Biol 12: 90–103. 21252998 10.1038/nrm3047 PMC3905242

[32] Alani E, Thresher R, Griffith JD, Kolodner RD (1992) Characterization of DNA-binding and strand-exchange stimulation properties of y-RPA, a yeast single-strand-DNA-binding protein. J Mol Biol 227: 54–71. 1522601 10.1016/0022-2836(92)90681-9

[33] Sung P (1994) Catalysis of ATP-dependent homologous DNA pairing and strand exchange by yeast RAD51 protein. Science 265: 1241–1243. 8066464 10.1126/science.8066464

[34] Solinger JA, Kiianitsa K, Heyer WD (2002) Rad54, a Swi2/Snf2-like recombinational repair protein, disassembles Rad51:dsDNA filaments. Mol Cell 10: 1175–1188. 12453424 10.1016/s1097-2765(02)00743-8

[35] Byrne AB, Weirauch MT, Wong V, Koeva M, Dixon SJ, et al (2007) A global analysis of genetic interactions in Caenorhabditis elegans. J Biol 6: 8. 17897480 10.1186/jbiol58 PMC2373897

[36] Zhou BB, Bartek J (2004) Targeting the checkpoint kinases: chemosensitization versus chemoprotection. Nat Rev Cancer 4: 216–225. 14993903 10.1038/nrc1296

[37] Kim ST, Lim DS, Canman CE, Kastan MB (1999) Substrate specificities and identification of putative substrates of ATM kinase family members. J Biol Chem 274: 37538–37543. 10608806 10.1074/jbc.274.53.37538

[38] Bernstein KA, Reid RJ, Sunjevaric I, Demuth K, Burgess RC, et al (2011) The Shu complex, which contains Rad51 paralogues, promotes DNA repair through inhibition of the Srs2 anti-recombinase. Mol Biol Cell 22: 1599–1607. 21372173 10.1091/mbc.E10-08-0691 PMC3084681

[39] Martin V, Chahwan C, Gao H, Blais V, Wohlschlegel J, et al (2006) Sws1 is a conserved regulator of homologous recombination in eukaryotic cells. EMBO J 25: 2564–2574. 16710300 10.1038/sj.emboj.7601141 PMC1478202

[40] Youds JL, O'Neil NJ, Rose AM (2006) Homologous recombination is required for genome stability in the absence of DOG-1 in Caenorhabditis elegans. Genetics 173: 697–708. 16547095 10.1534/genetics.106.056879 PMC1526509

[41] Youds JL, Barber LJ, Ward JD, Collis SJ, O'Neil NJ, et al (2008) DOG-1 is the Caenorhabditis elegans BRIP1/FANCJ homologue and functions in interstrand cross-link repair. Mol Cell Biol 28: 1470–1479. 18086896 10.1128/MCB.01641-07 PMC2258786

[42] Gomez-Gonzalez B, Felipe-Abrio I, Aguilera A (2009) The S-phase checkpoint is required to respond to R-loops accumulated in THO mutants. Mol Cell Biol 29: 5203–5213. 19651896 10.1128/MCB.00402-09 PMC2747986

[43] Castellano-Pozo M, Garcia-Muse T, Aguilera A (2012) R-loops cause replication impairment and genome instability during meiosis. EMBO Rep 13: 923–929. 22878416 10.1038/embor.2012.119 PMC3463965

[44] Bienko M, Green CM, Crosetto N, Rudolf F, Zapart G, et al (2005) Ubiquitin-binding domains in Y-family polymerases regulate translesion synthesis. Science 310: 1821–1824. 16357261 10.1126/science.1120615

[45] Roerink SF, Koole W, Stapel LC, Romeijn RJ, Tijsterman M (2012) A broad requirement for TLS polymerases eta and kappa, and interacting sumoylation and nuclear pore proteins, in lesion bypass during C. elegans embryogenesis. PLoS Genet 8: e1002800. 22761594 10.1371/journal.pgen.1002800 PMC3386174

[46] Kai M, Wang TS (2003) Checkpoint activation regulates mutagenic translesion synthesis. Genes Dev 17: 64–76. 12514100 10.1101/gad.1043203 PMC195967

[47] Parrilla-Castellar ER, Arlander SJ, Karnitz L (2004) Dial 9-1-1 for DNA damage: the Rad9-Hus1-Rad1 (9-1-1) clamp complex. DNA Repair (Amst) 3: 1009–1014. 15279787 10.1016/j.dnarep.2004.03.032

[48] Kemp M, Sancar A (2009) DNA distress: just ring 9-1-1. Curr Biol 19: R733–734. 19833574 10.1016/j.cub.2009.07.026

[49] Helt CE, Wang W, Keng PC, Bambara RA (2005) Evidence that DNA damage detection machinery participates in DNA repair. Cell Cycle 4: 529–532. 15876866 10.4161/cc.4.4.1598

[50] Kim JW, Fukukawa C, Ueda K, Nishidate T, Katagiri T, et al (2010) Involvement of C12orf32 overexpression in breast carcinogenesis. Int J Oncol 37: 861–867. 20811708 10.3892/ijo_00000737

[51] Brenner S (1974) The genetics of Caenorhabditis elegans. Genetics 77: 71–94. 4366476 10.1093/genetics/77.1.71 PMC1213120

[52] Dernburg AF, McDonald K, Moulder G, Barstead R, Dresser M, et al (1998) Meiotic recombination in C. elegans initiates by a conserved mechanism and is dispensable for homologous chromosome synapsis. Cell 94: 387–398. 9708740 10.1016/s0092-8674(00)81481-6

[53] Zalevsky J, MacQueen AJ, Duffy JB, Kemphues KJ, Villeneuve AM (1999) Crossing over during Caenorhabditis elegans meiosis requires a conserved MutS-based pathway that is partially dispensable in budding yeast. Genetics 153: 1271–1283. 10545458 10.1093/genetics/153.3.1271 PMC1460811

[54] Hodgkin J, Horvitz HR, Brenner S (1979) Nondisjunction Mutants of the Nematode CAENORHABDITIS ELEGANS. Genetics 91: 67–94. 17248881 10.1093/genetics/91.1.67 PMC1213932

[55] Fares H, Greenwald I (1999) SEL-5, a serine/threonine kinase that facilitates lin-12 activity in Caenorhabditis elegans. Genetics 153: 1641–1654. 10581273 10.1093/genetics/153.4.1641 PMC1460874

[56] D'Agostino I, Merritt C, Chen PL, Seydoux G, Subramaniam K (2006) Translational repression restricts expression of the C. elegans Nanos homolog NOS-2 to the embryonic germline. Dev Biol 292: 244–252. 16499902 10.1016/j.ydbio.2005.11.046

[57] Merritt C, Rasoloson D, Seydoux G (2010) Transgenic solutions for the germline, Wormbook.1.148.1. 10.1895/wormbook.1.148.1 PMC4966531 20169625

[58] Kage-Nakadai E, Kobuna H, Funatsu O, Otori M, Gengyo-Ando K, et al (2012) Single/low-copy integration of transgenes in Caenorhabditis elegans using an ultraviolet trimethylpsoralen method. BMC Biotechnol 12: 1. 22217006 10.1186/1472-6750-12-1 PMC3262153

[59] Sonnhammer EL, Eddy SR, Durbin R (1997) Pfam: a comprehensive database of protein domain families based on seed alignments. Proteins 28: 405–420. 9223186 10.1002/(sici)1097-0134(199707)28:3<405::aid-prot10>3.0.co;2-l

[60] Timmons L, Court DL, Fire A (2001) Ingestion of bacterially expressed dsRNAs can produce specific and potent genetic interference in Caenorhabditis elegans. Gene 263: 103–112. 11223248 10.1016/s0378-1119(00)00579-5

[61] Kim SH, Holway AH, Wolff S, Dillin A, Michael WM (2007) SMK-1/PPH-4.1-mediated silencing of the CHK-1 response to DNA damage in early C. elegans embryos. J Cell Biol 179: 41–52. 17908915 10.1083/jcb.200705182 PMC2064732

[62] MacQueen AJ, Colaiacovo MP, McDonald K, Villeneuve AM (2002) Synapsis-dependent and -independent mechanisms stabilize homolog pairing during meiotic prophase in C. elegans. Genes Dev 16: 2428–2442. 12231631 10.1101/gad.1011602 PMC187442

[63] Walhout AJ, Vidal M (2001) High-throughput yeast two-hybrid assays for large-scale protein interaction mapping. Methods 24: 297–306. 11403578 10.1006/meth.2001.1190

[64] Vidal M, Brachmann RK, Fattaey A, Harlow E, Boeke JD (1996) Reverse two-hybrid and one-hybrid systems to detect dissociation of protein-protein and DNA-protein interactions. Proc Natl Acad Sci U S A 93: 10315–10320. 8816797 10.1073/pnas.93.19.10315 PMC38381

