# Supplementary figures and images for: ZTF-8 Interacts with the 9-1-1 Complex and Is Required for DNA Damage Response and Double-Strand Break Repair in the C. elegans Germline
Source: PLoS Genet. 2014 Oct 16;10(10):e1004723. doi: 10.1371/journal.pgen.1004723 (PMC4199516; doi:10.1371/journal.pgen.1004723)

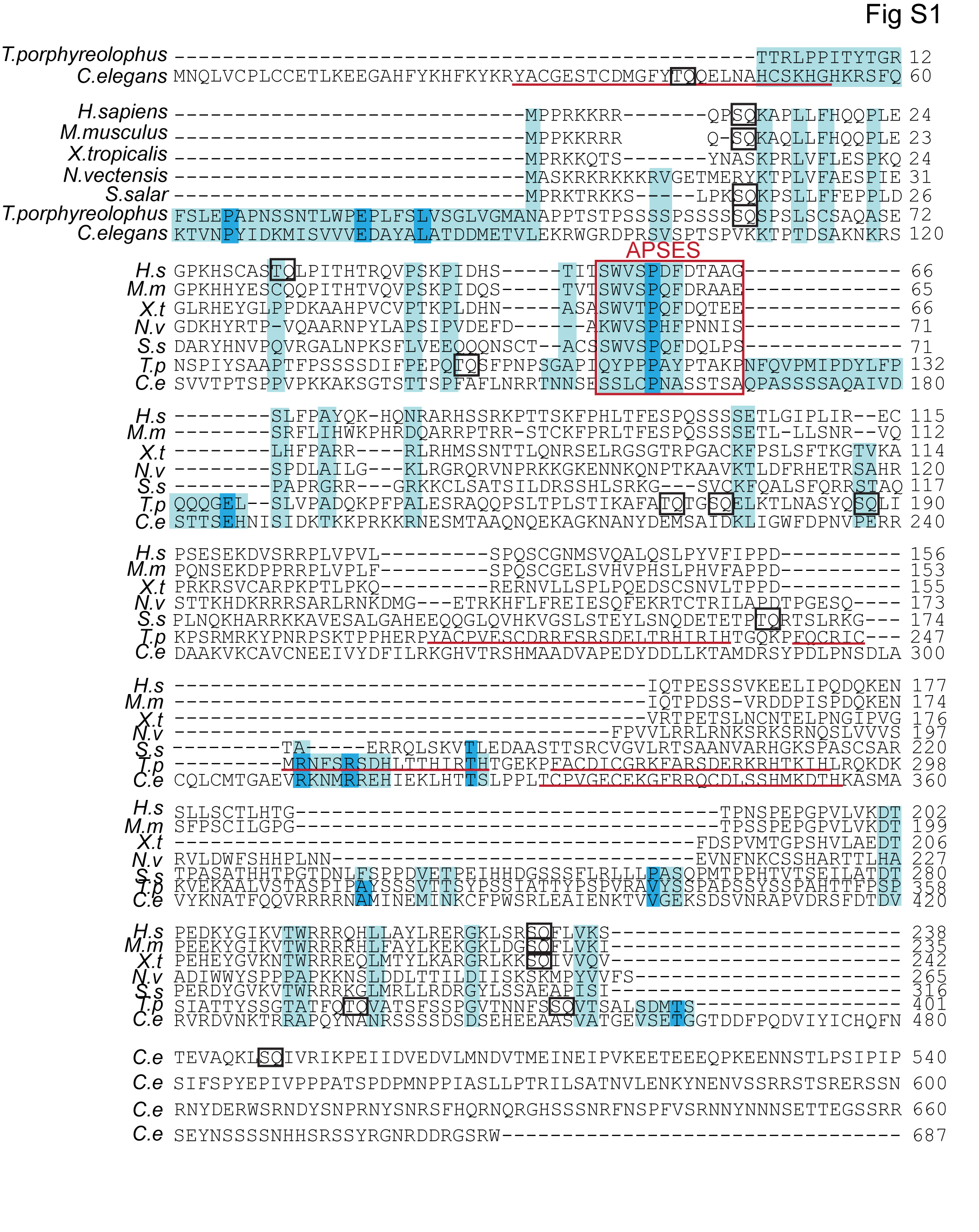

Supplement: Figure S1 — ZTF-8 protein conservation. Sequence alignment between C. elegans ZTF-8 and its predicted homologs in H. sapiens, M. musculus, X. tropicalis, N. vectensis, S. Salar, and T. porphyreolophus. Alignment was performed using CLUSTAL 2.1 from EMBL-EBI (www.ebi.ac.uk) and Pfam (http://pfam.sanger.ac.uk). Shaded dark blue boxes indicate amino acid identity and light blue boxes indicate similarity. 8% identity and 15% of amino acid sequence similarity was found between RHINO (H. sapiens) and ZTF-8 (C. elegans) by using CLUSTAL 2.1. Zinc-finger motifs were identified using Prosite (http://prosite.expasy.org) and are underlined with red lines. A red-colored box indicates the hypothetical APSES DNA binding site found in different species. Black-colored boxes indicate SQ and TQ sites. (TIF) [file pgen.1004723.s001.tif]

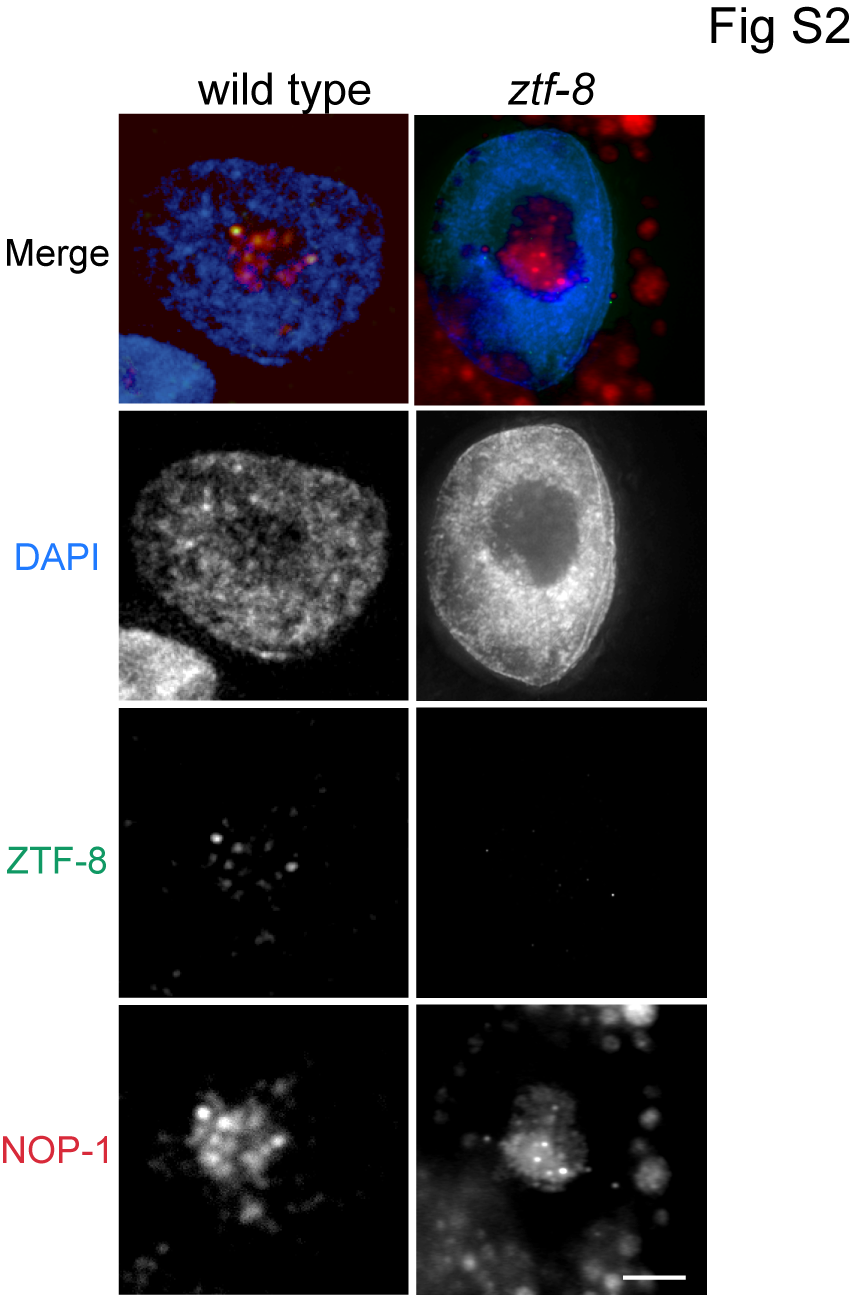

Supplement: Figure S2 — ZTF-8 localization to somatic nuclei. Co-staining of intestinal nuclei from wild type and ztf-8 mutants with DAPI (blue), an anti-NOP-1 antibody (red) and an anti-ZTF-8 antibody (green). Bar, 2 µm. (TIF) [file pgen.1004723.s002.tif]

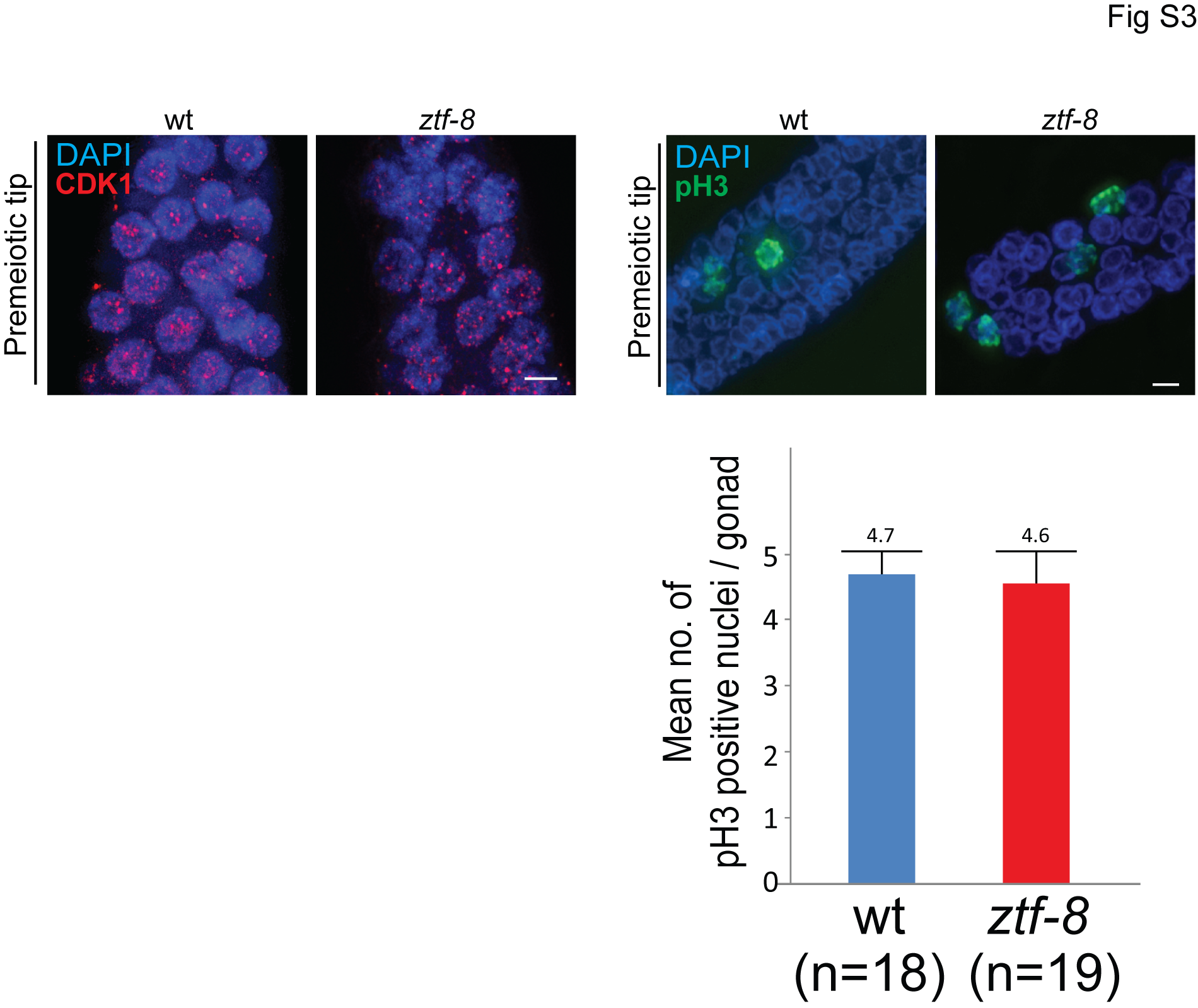

Supplement: Figure S3 — Assessing mitotic progression by immunostaining with G2/M markers. Immunostaining of wild type and ztf-8 mutants with DAPI and an anti CDK1 pTyr15 antibody or an anti phospho-histone H3 pSer10 antibody. ztf-8 mutants exhibit similar staining pattern in either CDK-1 (n = 8 germlines for both genotypes) or pH 3 staining (n = 18 for wild type and n = 19 for ztf-8 mutants, P = 0.9139 by the two-tailed Mann-Whitney test, 95% C.I.). Bar, 2 µm. (TIF) [file pgen.1004723.s003.tif]

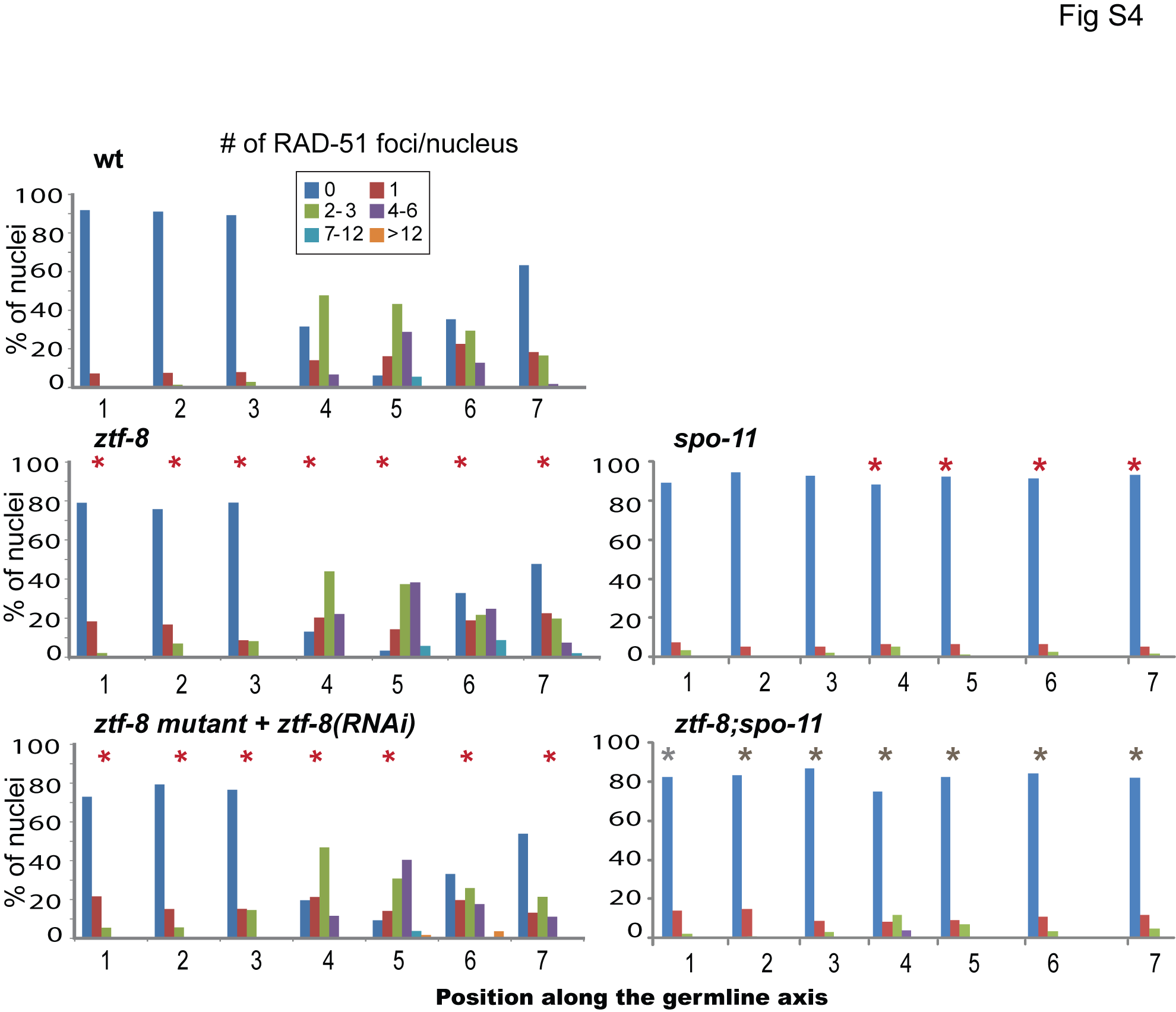

Supplement: Figure S4 — Assessing DSBR progression by quantitation of RAD-51 foci. Graphs depict the percentage of nuclei carrying RAD-51 foci (y-axis) within each zone along the germline (x-axis). Asterisks indicate statistical significance compared to either wild type (*) or spo-11 (*). (TIF) [file pgen.1004723.s004.tif]

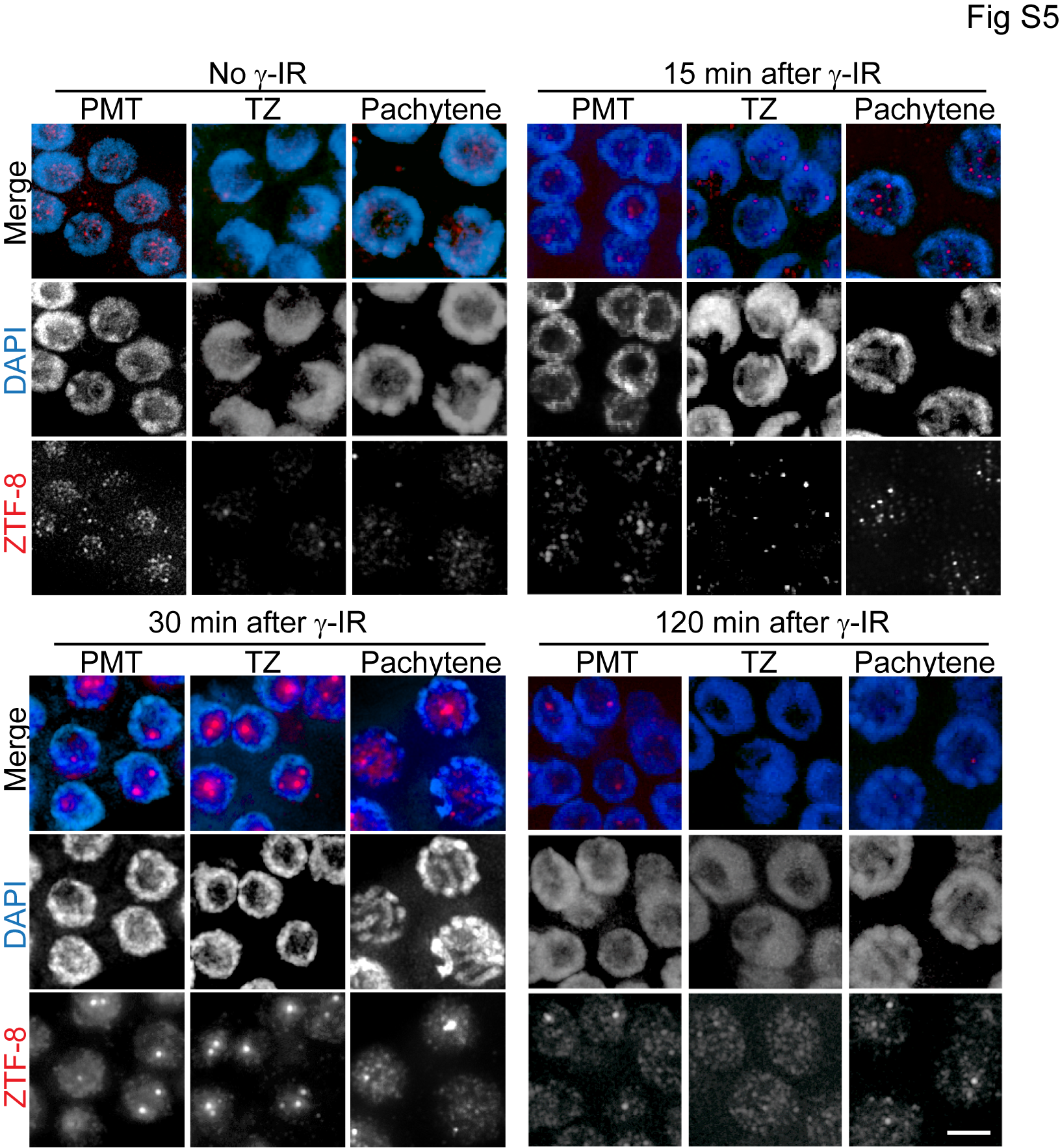

Supplement: Figure S5 — ZTF-8 localization changes in response to exogenous DSB formation. Immunolocalization of ZTF-8 prior to and 15, 30 and 120 minutes following γ-IR exposure (50 Gy). PMT, premeiotic tip; TZ, transition zone. Bar, 2 µm. (TIF) [file pgen.1004723.s005.tif]

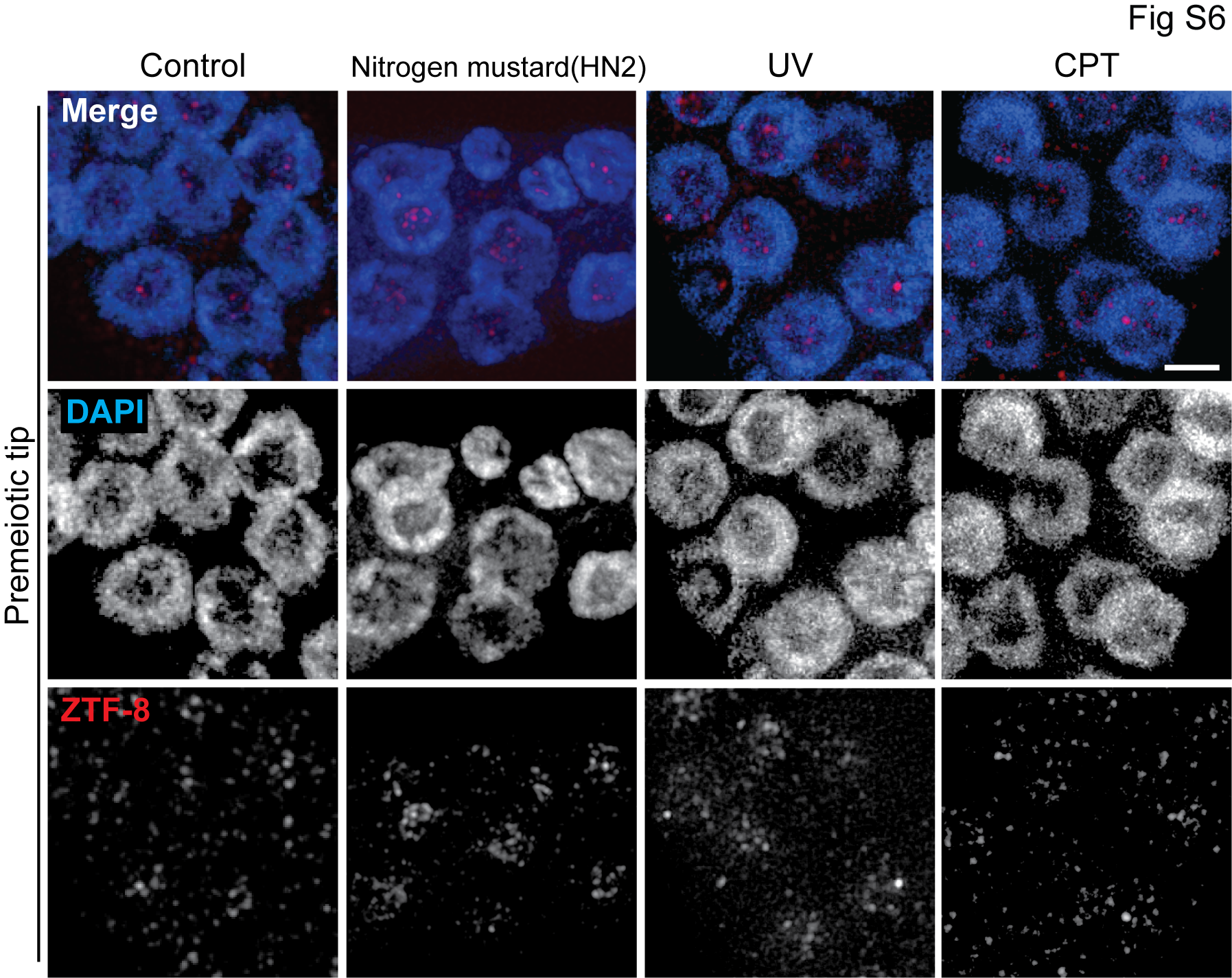

Supplement: Figure S6 — ZTF-8 localization does not change in response to HN2, UV and CPT treatment. Immunolocalization of ZTF-8 30 minutes after exposure to UVC (150 J/m2), CPT (500 nM), and HN2 (150 µM). Control contains DMSO only. Bar, 2 µm. (TIF) [file pgen.1004723.s006.tif]
