# Supplementary material for: ZTF-8 Interacts with the 9-1-1 Complex and Is Required for DNA Damage Response and Double-Strand Break Repair in the C. elegans Germline
Source: PLoS Genet. 2014 Oct 16;10(10):e1004723. doi: 10.1371/journal.pgen.1004723 (PMC4199516; doi:10.1371/journal.pgen.1004723)
Supplement: Table S1 — Primers used for the yeast two-hybrid experiments. These primers were utilized to generate the full length and truncations of ZTF-8. (DOC) [file pgen.1004723.s007.doc]

**Table S1. Primers used for the yeast two-hybrid experiments**

| **Primer name** | **Primer sequence** |
| --- | --- |
| ZTF-8-full length-F | AACCAATTAGTGTGTCC |
| ZTF-8-full length-R | TTACCATCGAGAGCCTCGATCATC |
| ZTF-8-C-Ter-F | GGGGAGAAGTCGGATTCTGTTAAC |
| ZTF-8-C-Ter-R | TTACCATCGAGAGCCTCGATCATC |
| ZTF-8-N-Ter*-*F | AACCAATTAGTGTGTCC |
| ZTF-8-N-Ter*-*R | TGTTAGCGGTGGAAGACTCGTCGT |
| ZTF-8-M-F | GCAGCTGATGTAGCACCAGAAGA |
| ZTF-8-M-R | GGAAGAACGTTCACGTGATGTCGATCT |
|  |  |
